# Endothelial biomarkers predict kidney and cardiovascular outcomes after acute kidney injury

**DOI:** 10.1093/ckj/sfag277

**Published:** 2026-08-13

**Authors:** Giovana S Di Marco, Katrin Beul, Eike Bormann, Hermann Pavenstädt, Marcus Brand

**Affiliations:** Department of Internal Medicine D, University Hospital Muenster, Münster, Germany; Department of Internal Medicine D, University Hospital Muenster, Münster, Germany; Institute for Biostatistics and Clinical Research, University Hospital Münster, Muenster, Germany; Department of Internal Medicine D, University Hospital Muenster, Münster, Germany; Department of Internal Medicine D, University Hospital Muenster, Münster, Germany

**Keywords:** acute kidney injury, cardiovascular disease, CD138, endothelial damage, renal nonrecovery, soluble fms-like tyrosine kinase receptor (sFlt-1)

## Abstract

**Background:**

Acute kidney injury (AKI) is common in hospitalized patients, and post-injury biological processes activated after the initial insult may determine recovery or sustained damage. Identifying these processes may offer opportunities for early intervention, and biomarkers can serve as valuable tools in this context. We hypothesized that endothelial biomarkers—reflecting endothelial dysfunction or damage—predict adverse clinical outcomes following AKI.

**Methods:**

In a single-center cohort of 93 consecutive patients with in-hospital mild AKI, a panel of endothelial (sFlt-1, CD138, and sVCAM-1) and renal stress (IGFBP7, TIMP2, and their product [IGFBP7]*[TIMP2]) biomarkers was measured at diagnosis. Associations with in-hospital mortality, AKI duration ≥7 days, new-onset cardiovascular disease (CVD), and renal nonrecovery were examined using regression analyses.

**Results:**

Endothelial biomarkers were more closely associated with adverse outcomes than renal stress biomarkers in this cohort. After adjustment, each 100-unit increase in sFlt-1 was independently associated with higher odds of in-hospital death (OR 1.034 [1.007–1.062], *P *= 0.0144), new-onset CVD (OR 1.068 [1.029–1.108], *P *= 0.0005), and renal nonrecovery (OR 1.158 [1.045–1.284], *P *= 0.0052). Similarly, each 10-unit increase in CD138 was associated with increased odds of in-hospital mortality (OR 1.050 [1.014–1.088], *P *= 0.006) and new-onset CVD (OR 1.040 [1.004–1.077], *P *= 0.0304). Inclusion of endothelial biomarkers improved predictive performance for renal nonrecovery (AUC 0.86 vs. 0.75, *P *= 0.0163) and new-onset CVD (AUC 0.84 vs. 0.69, *P *= 0.0059).

**Conclusions:**

Endothelial biomarkers, particularly sFlt-1 and CD138, provide insight into vascular health following AKI and independently predict clinical outcomes. Their integration into clinical models may improve risk stratification, supporting personalized post-AKI care strategies.

## Graphical Abstract

**Graphical Abstract:**
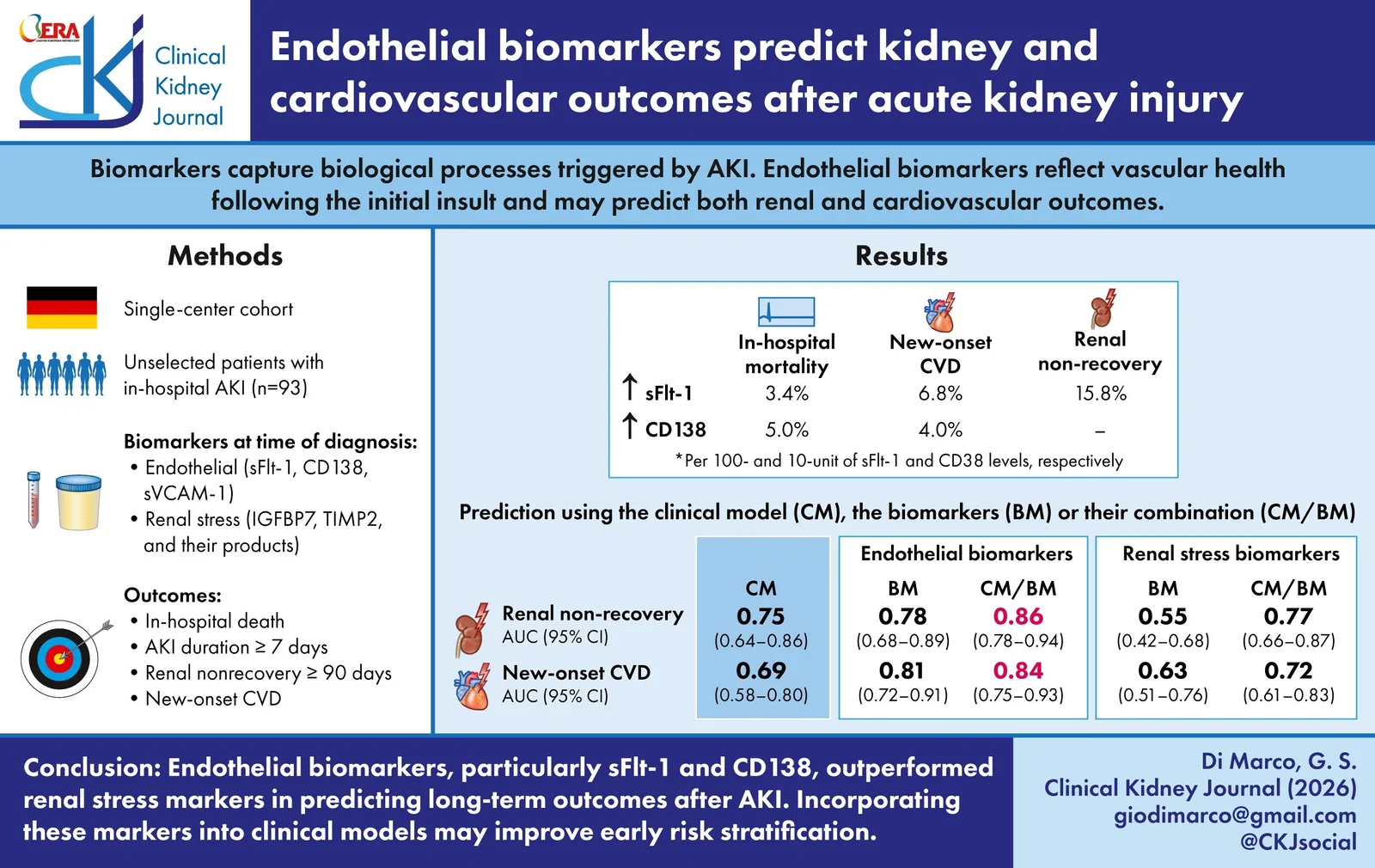

KEY LEARNING POINTS **What was known:**

- Endothelial biomarkers are associated with adverse outcomes after AKI, independent of its cause.
- sFlt-1 and CD138 have shown prognostic value in selected populations, such as post-cardiac surgery and kidney transplant patients.
- However, their predictive role in broader and less severe AKI populations remained unclear.

**This study adds**:

- sFlt-1 and CD138 measured at AKI diagnosis are strong predictors of in-hospital mortality and new-onset cardiovascular disease.
- sFlt-1 is also independently associated with renal nonrecovery in non-critically ill hospitalized patients with mild AKI.
- Endothelial biomarkers provided complementary prognostic information beyond renal stress markers.

**Potential impact:**

- Incorporating endothelial biomarkers into clinical models may improve early risk stratification in AKI.
- This could guide individualized monitoring and intervention strategies during hospitalization and post-discharge.
- Ultimately, this approach may help reduce long-term cardiovascular and renal complications in AKI survivors.

## INTRODUCTION

Acute kidney injury (AKI) is common in hospitalized patients, affecting 10%–15% of admissions. Most published studies on AKI have focused on critically ill patients admitted to intensive care units (ICUs), in whom sepsis represents the predominant driver of dysfunction. However, recent epidemiologic data indicate that AKI is also a frequent complication in low- and medium-intensity care settings [1, 2]. Importantly, emerging evidence suggests that even mild AKI, including low-stage and rapidly recovering AKI, may still be associated with adverse clinical consequences [3–5]. AKI-associated complications include chronic kidney disease and cardiovascular disease (CVD) [6, 7], with incomplete recovery after AKI representing a major driver of these adverse outcomes [2].

After an initial insult, the kidneys may either undergo adaptive repair and regeneration or maladaptive repair, leading to fibrosis and progression to chronic disease [8–10]. Early identification of the biological processes driving these divergent pathways is essential, as it enables clinicians to target treatments and preventive measures for high-risk individuals, potentially improving survival and quality of life [9–11].

Biomarkers are valuable tools to capture such processes and define AKI subphenotypes with distinct prognoses. For example, urinary insulin-like growth factor-binding protein-7 (IGFBP7) and tissue inhibitor of metalloproteinases-2 (TIMP2) reflect tubular stress and damage [12–14]. Notably, elevated urinary IGFBP7 and TIMP2 levels on the first day of AKI diagnosis have been shown to predict renal nonrecovery [15, 16].

Endothelial biomarkers also provide critical insights, as endothelial health is crucial for kidney function, adaptive repair, and cardiovascular health [17, 18]. These biomarkers can further signal cardiovascular dysfunction and long-term chronic disease risks [17]. Syndecan-1 (CD138) reflects endothelial glycocalyx (eGC) degradation [19, 20], while soluble Fms-like tyrosine kinase 1 (sFlt-1) is linked to both vascular endothelial growth factor antagonism and eGC disruption [21, 22]. Emerging evidence suggests that endothelial dysfunction-associated AKI subphenotypes are strongly tied to adverse outcomes [8, 9, 11].

We therefore hypothesized that endothelial biomarkers predict renal nonrecovery and other adverse outcomes in non-critically ill hospitalized patients with AKI. By focusing on this understudied but clinically relevant subgroup, we aimed to reduce confounding from systemic inflammation and multiorgan dysfunction, which can independently affect endothelial biomarker levels.

## MATERIALS AND METHODS

Extended methodological details regarding population description, sampling procedures, and outcome definitions are provided in the Supplementary Methods.

### Settings and study population

This longitudinal study included 93 consecutive adult patients diagnosed with AKI and treated at the University Hospital Münster between August 2021 and July 2023. Ethical approval was obtained from the Joint Ethics Committee of the Landesärztekammer Westfalen-Lippe and the University of Münster (No. 2021-470-f-S), and all patients provided written informed consent.

Patients were identified via the hospital’s electronic records according to KDIGO criteria: serum creatinine (sCrea) increase ≥0.3 mg/dl within 48 h or ≥1.5-fold from baseline within 7 days. Baseline sCrea was defined as the admission or the closest outpatient value. At AKI diagnosis, defined as the first time point at which KDIGO criteria fulfillment was confirmed by a nephrologist, urine and blood samples were collected. AKI stage was assigned per KDIGO. Exclusion criteria: age <18 years, missing consent, sepsis, admission to the ICU, SARS-CoV-2 infection, stage 5 CKD/dialysis, organ transplantation, malignancy, pregnancy, or breastfeeding. Exclusion criteria: age <18 years, missing consent, sepsis, admission to the ICU, SARS-CoV-2 infection, stage 5 CKD/dialysis, organ transplantation, malignancy, pregnancy, or breastfeeding. Critically ill patients (e.g. patients with sepsis or requiring intensive care) were excluded to reduce confounding from systemic inflammation and multiorgan dysfunction, both of which substantially influence endothelial biomarker concentrations independent of AKI-specific mechanisms [23–27].

**Figure 1:**
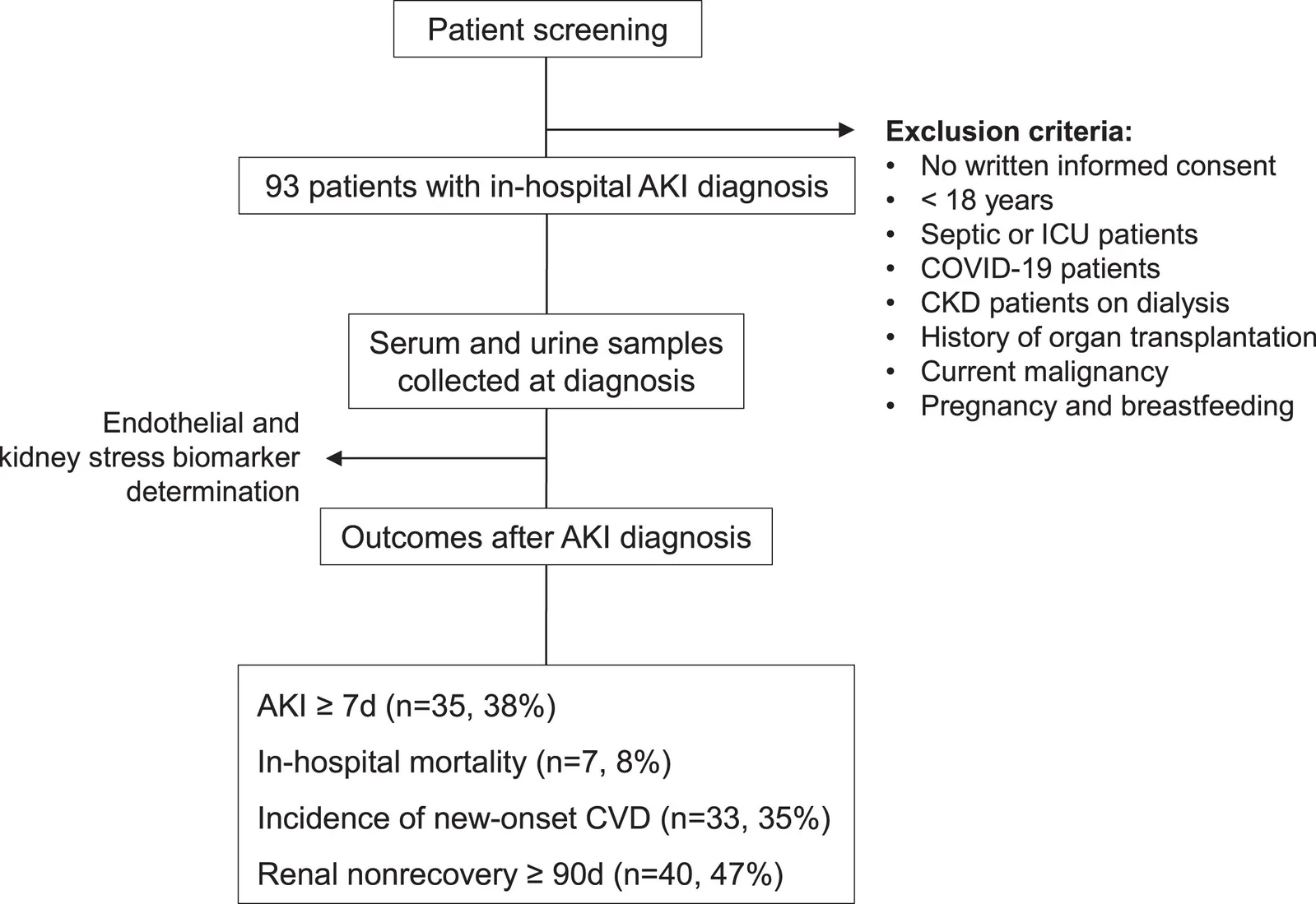
Flowchart of the study. AKI, acute kidney injury; CKD, chronic kidney disease; COVID-19, coronavirus disease; CVD, cardiovascular disease; and ICU, intensive care unit. Patients with significant changes in sCrea were identified through the electronic medical record system and evaluated by a nephrologist. Once KDIGO criteria were met and exclusion criteria were absent, patients were enrolled and samples were collected immediately. The follow-up period ranged from 3 to 7 months after AKI diagnosis.

Primary outcomes were AKI duration, in-hospital mortality, new-onset CVD, and renal nonrecovery ≥90 days post-AKI, defined as eGFR <90% of baseline. Figure 1 shows patient enrollment and study design.

### Data collection and laboratory analyses

Detailed demographic, laboratory, and clinical data were obtained from a computerized database. All GFR values were estimated using the CKD-EPI equation.

Serum and urinary biomarkers were measured using commercial ELISA kits. Urinary biomarkers were normalized to creatinine. The [IGFBP7]*[TIMP2] product was calculated by multiplying IGFBP7 and TIMP2 concentrations (ng/ml) and dividing by 1000 [15].

### Statistical analysis

Categorial variables are presented as absolute and relative frequencies. Continuous variables are presented as mean and standard deviation for normally distributed variables, or as median with 25th and 75th percentiles for non-normally distributed variables. Fisher’s exact test was used to compare categorical variables between groups. Analysis of variance (ANOVA) was used to compare normally distributed continuous variables across three groups, and the Kruskal–Wallis test was used for non-normally distributed continuous variables. Spearman’s rank correlation coefficient was used to evaluate the correlation among the biomarkers sFlt-1, CD138, serum vascular cell adhesion molecule-1 (sVCAM-1), IGFBP7, TIMP2, and [IGFBP7]*[TIMP2], as well as between the biomarkers and the variables: sCrea, baseline eGFR, C-reactive protein (CRP), proteinuria, change in sCrea, duration of AKI, length of hospitalization, and follow-up eGFR.

Continuous biomarkers were modeled in increments that were clinically meaningful (100 units for sFlt-1 and 10 units for CD138), based on their observed distributions, to enhance the interpretability of regression estimates. Tertiles of sFlt-1 were used exclusively for descriptive analyses, and stratification by tertiles was performed for sFlt-1, the biomarker with the strongest association with outcome variables in preliminary analyses. KDIGO stage was included as a covariate in all adjusted models to account for AKI severity. Univariate logistic regression was used to evaluate the influence of sFlt-1, CD138, and sVCAM-1 on AKI duration ≥7 days, in-hospital mortality, new-onset CVD, and renal nonrecovery. Additionally, a multivariable logistic regression model was used to evaluate the effects of sFlt-1, CD138, and sVCAM-1 on AKI duration ≥7 days, new-onset CVD and renal nonrecovery, adjusting for age, sex, baseline eGFR, history of hypertension, diabetes mellitus, or CVD, change in sCrea from baseline to diagnosis, AKI stage, proteinuria, body mass index, CRP, IGFBP7, TIMP2, and [IGFBP7]*[TIMP2]. For in-hospital mortality, only sFlt-1, CD138, sVCAM-1, IGFBP7, TIMP2, and [IGFBP7]*[TIMP2] were included as independent variables due to the low number of events, which limits the stability of multivariable models and the reliability of effect estimates. To account for heterogeneity of AKI pathophysiology, additional multivariable logistic regression models were constructed including AKI etiology as covariates. Because the number of patients within individual etiologic categories was limited, only the three major AKI mechanisms—postoperative, nephrotoxic, and perfusion-related AKI—were included. To minimize overfitting, these analyses were performed using a parsimonious set of clinically relevant covariates, including age, sex, baseline eGFR, and AKI severity. Receiver operating curves (ROC) were used to compare different models. Differences between ROCs were evaluated using the DeLong test.

## RESULTS

### Patients’ characteristics

Among the 93 patients who developed AKI during their hospital stay, the median age was 69 years (interquartile range, 61–78), and 41 (44%) were female. Tables 1 and 2 summarize the main patient characteristics and clinical outcomes. Stratification by sFlt-1 tertiles was selected because sFlt-1 showed the strongest association with adverse outcomes among the endothelial biomarkers studied. Twenty-four patients (26%) presented with unifactorial AKI, while 69 patients (74%) had two or more concurrent contributing causes of AKI, consistent with a multifactorial etiology. Table 1 shows the distribution of all AKI causes in the total population, whereas Supplementary Table S1 depicts the causes stratified by uni- and multifactorial AKI according to serum sFlt-1 levels. The most frequent contributors to multifactorial AKI were perfusion-related factors, postoperative complications, and exposure to nephrotoxic drugs, present in 51 (74%), 27 (39%), and 26 (38%) patients, respectively. Among the patients with a single identified cause of AKI, nephrotoxicity, surgery, and obstruction accounted for 8 (9%), 5 (5%), and 4 (4%) cases, respectively. No statistically significant differences in the distribution of AKI causes were observed across serum sFlt-1 tertiles. Supplementary Table S2 presents the primary reasons for hospitalization according to serum sFlt-1 levels. All patients had their prescribed medications adjusted according to renal function immediately after the AKI diagnosis. KDIGO stage 1 was most common, affecting 56 patients (60%), followed by stage 2 AKI in 23 patients (25%), while only 14 patients (15%) had stage 3 disease (Table 1). Thirty-five patients (38%) experienced persistent AKI (≥7 days of duration), and 7 (8%) died during hospitalization. Among the survivors, 40 (47%) patients did not recover renal function ≥90 days after diagnosis. Over the entire follow-up period, 33 patients (35%) developed new-onset CVD. Three patients died at 1.9, 6.9, and 9.9 months after diagnosis. The average follow-up time was 5 ± 2 months (Table 2).

**Table 1:** Patients’ characteristics according to serum sFlt-1 levels.

		Tertiles of sFlt-1 (pg/ml)			
	Total	First	Second	Third	*P*-Value^1^
Total/survivors	*n* = 93/85	*n* = 31/29	*n* = 31/31	*n* = 31/26	
***Endothelial biomarkers***					
sFlt-1 (*n* = 93)	201 (20–1036)	10 (8–20)	201 (127–474)	2429 (1036–5258)	.0000
CD138 (*n* = 93)	68 (42–103)	49 (28–82)	55 (39–99)	97 (71–276)	.0003
sVCAM-1 (*n* = 93)	1402 ± 630	1473 ± 632	1352 ± 562	1380 ± 704	.7334
***Patient demographics***					
Age	69 (61–78)	73 (65–80)	70 (59–79)	66 (56–71)	.0428
Sex					.0249
*Female*	41 (44)	20 (65)	10 (32)	11 (35)	
*Male*	52 (56)	11 (35)	21 (68)	20 (65)	
***Clinical data***					
CVD	46 (49)	16 (52)	17 (55)	13 (42)	.6554
Diabetes	34 (37)	14 (45)	12 (39)	8 (26)	.3225
Hypertension	64 (69)	21 (68)	22 (71)	21 (68)	1.
DBP^2^	67 ± 14	70 ± 13	64 ± 12	68 ± 18	.3134
SBP^2^	120 ± 20	124 ± 23	121 ± 18	116 ± 19	.3199
BMI	25 (23–27)	25 (23–27)	25 (24–27)	26 (23–29)	.8041
Hb	11 ± 3	11 ± 3	10 ± 3	10 ± 3	.5967
CRP	4.2 (1.6–10.1)	3.7 (1.4–7.4)	7.1 (2.0–13.4)	4.1 (2.0–8.6)	.1916
Leukocytes	9.7 (7.0–12.2)	10.5 (6.7–13.1)	9.7 (8.0–12.5)	9.3 (7.0–11.4)	.5523
BUN^3^	36 (26–53)	32 (22–40)	45 (27–57)	35 (27–62)	.1352
Proteinuria^4^	0.6 (0.3–1.7)	0.8 (0.2–1.7)	0.5 (0.4–1.2)	0.8 (0.3–2.0)	.8514
Baseline eGFR	58 ± 24	58 ± 21	56 ± 26	60 ± 25	.7854
Diagnosis eGFR	29 (18–39)	33 (19–41)	25 (15–37)	30 (18–39)	.7036
Baseline sCrea	1.2 (0.9–1.5)	1.1 (0.9–1.4)	1.3 (0.9–1.9)	1.2 (0.9–1.5)	.2092
Diagnosis sCrea	2.1 (1.6–3.0)	1.8 (1.5–2.6)	2.5 (1.8–3.2)	2.1 (1.8–2.7)	.1039
AKI stage					.1162
*Stage 1*	56 (60)	19 (61)	19 (61)	18 (58)	
*Stage 2*	23 (25)	11 (35)	5 (16)	7 (23)	
*Stage 3*	14 (15)	1 (3)	7 (23)	6 (19)	
***Causes of AKI***					
*Perfusion-related AKI^5^*	52 (56)	16 (52)	17 (55)	19 (61)	.8090
*Cardiorenal syndrome*	14 (15)	4 (13)	5 (16)	5 (16)	1.000
*Hepatorenal syndrome*	6 (7)	2 (6)	0 (0)	4 (13)	.1595
*Nephrotoxic AKI^6^*	34 (37)	12 (39)	14 (45)	8 (26)	.3225
*Rhabdomyolysis*	2 (2)	2 (6)	0 (0)	0 (0)	.3261
*Glomerular/interstitial AKI^7^*	4 (4)	3 (10)	1 (3)	0 (0)	.3187
*Hypercalcemia*	2 (2)	0 (0)	2 (6)	0 (0)	.3261
*Obstruction*	6 (6)	2 (6)	1 (3)	3 (10)	.8681
*Postoperative-AKI*	32 (34)	10 (32)	13 (42)	9 (29)	.6268
*Inflammation-associated AKI^8^*	15 (16)	5 (16)	5 (16)	5 (16)	1.000
*Multifactorial AKI^9^*	69 (74)	24 (77)	21 (67)	24 (77)	.7264
***Medication***					
*ACE inhibitors*	25 (27)	9 (29)	8 (26)	8 (26)	1.000
*AT1 blockers*	21 (23)	11 (35)	6 (19)	4 (13)	.1317
*NSAIDs*	10 (11)	4 (13)	2 (6)	4 (13)	.7639
*Ca^2+^ antagonists*	30 (32)	15 (48)	9 (29)	6 (19)	.0529
*Beta blockers*	51 (55)	19 (61)	16 (52)	16 (52)	.7068
*Diuretics*	51 (55)	20 (65)	18 (58)	13 (42)	.2208
***Renal biomarkers***					
Urine IGFPB7^4^	242 (163–385)	221 (171–316)	220 (148–363)	299 (160–588)	.5670
Urine TIMP2^4^	7 (4–10)	6 (4–8)	6 (4–8)	8 (5–12)	.2834
[IGFPB7]*[TIMP2]^4^	1.7 (0.8–4.4)	1.5 (0.8–2.6)	1.4 (0.8–3.3)	2.3 (0.8–6.8)	.3557

Numerical data are presented as mean ± SD for normally distributed variables or as median (IQR) for non-normally distributed variables. Categorical variables are shown as counts and percentages [*n* (%)]. ¹*P*-values were obtained using ANOVA, Kruskal–Wallis, or Fisher’s exact test, as appropriate. ^2^One value is missing (*n* = 92); ^3^Twenty-three values are missing (*n* = 70); ^4^Four urine samples are unavailable (*n* = 89), two of the four patients with missing urine samples had AKI stage 3 and were anuric; the others had AKI stages 2 and 1. ^5^AKI occurring in the setting of reduced effective renal blood flow, including hypovolemia, hypotension, and renal hypoperfusion; ^6^AKI associated with exposure to nephrotoxic drugs. ^7^AKI due to acute interstitial nephritis or glomerulonephritis. ^8^AKI temporally associated with systemic inflammatory conditions after exclusion of purely postrenal causes. ^9^Sixty-nine patients presented with two or more concurrent contributing causes (multifactorial AKI). Abbreviations and units: ACEi, angiotensin-converting enzyme inhibitors; Age, in years; AT_1_, angiotensin receptor 1, BMI, body mass index (kg/m^2^); BUN, blood uren nitrogen (mg/dl); CD138, syndecan-1 (pg/ml); sCrea, serum creatinine (mg/dl); CRP, C-reactive protein (mg/dl); CVD, cardiovascular disease; DBP, diastolic blood pressure (mmHg); sFlt-1, soluble Fms-like tyrosine kinase-1 (pg/ml); eGFR, estimated glomerular filtration rate (ml/min/1.73m^2^); IGFBP7; urinary insulin-like growth factor-binding protein 7 (ng/mg creatinine); [IGFBP7]*[TIMP-2] ((ng/ml)²/1000); Leukocytes, thousands per microliter; NSAIDs, nonsteroidal anti-inflammatory drugs; Proteinuria, mg protein/mg creatinine; SBP, systolic blood pressure (mmHg); TIMP2, urinary tissue inhibitor of metalloproteinase 2 (ng/mg creatinine); and sVCAM-1, soluble vascular cell adhesion molecule-1 (ng/ml). All urine and serum samples for biomarker determination were collected at the time of AKI diagnosis.

**Table 2:** Patient outcomes according to serum sFlt-1 levels.

		Tertiles of sFlt-1 (pg/ml)			
	Total	First	Second	Third	*P*-Value^1^
Total/survivors	*n* = 93/85	*n* = 31/29	*n* = 31/31	*n* = 31/26	
Change in sCrea	0.8 (0.5–1.4)	0.7 (0.5–1.3)	0.70 (0.5–1.5)	0.80 (0.6–1.8)	.7350
Duration of AKI	5 (3–8)	4 (2–6)	5 (3–9)	7 (4–12)	.0094
AKI ≥ 7 days	35 (38)	6 (19)	13 (42)	16 (52)	.0264
Length of hospitalization	9 (5–15)	8 (4–11)	8 (6–20)	11 (6–17)	.2331
In-hospital death	7 (8)	2 (6)	0 (0)	5 (16)	.0653
Renal nonrecovery^2^	40 (47)	6 (21)	13 (43)	21 (81)	<0.0001
Follow-up eGFR^2^	49 ± 23	63 ± 18	47 ± 21	35 ± 21	.0000
New-onset CVD	33 (35)	4 (13)	8 (26)	21 (68)	<0.0001
Length of follow-up^2^	5 ± 2	5 ± 2	6 ± 2	5 ± 2	.7727

Numerical data are presented as mean ± SD for normally distributed variables or as median (IQR) for non-normally distributed variables. Categorical variables are shown as counts and percentages [*n* (%)]. ¹*P*-values were obtained using ANOVA, Kruskal–Wallis, or Fisher’s exact test, as appropriate. ^2^Outcomes assessed in survivors (*n* = 85) ≥90 days after AKI diagnosis. Abbreviations and units: sCrea, serum creatinine (mg/dl); CVD, cardiovascular disease; duration of AKI, in days; sFlt-1, soluble Fms-like tyrosine kinase-1 (pg/ml); length of follow-up, in days; eGFR, estimated glomerular filtration rate (ml/min/1.73 m^2^); length of hospitalization, in days. All urine and serum samples for biomarker determination were collected at the time of AKI diagnosis.

In the subgroup of 4 patients with missing urine samples, two had AKI stage 3 and were anuric; and only one of them experienced renal nonrecovery. The other 2 patients had AKI stages 2 and 1. New-onset CVD occurred only in the patient with AKI stage 2, and none died during follow-up.

### Associations of endothelial and renal biomarkers with outcomes

**Figure 2:**
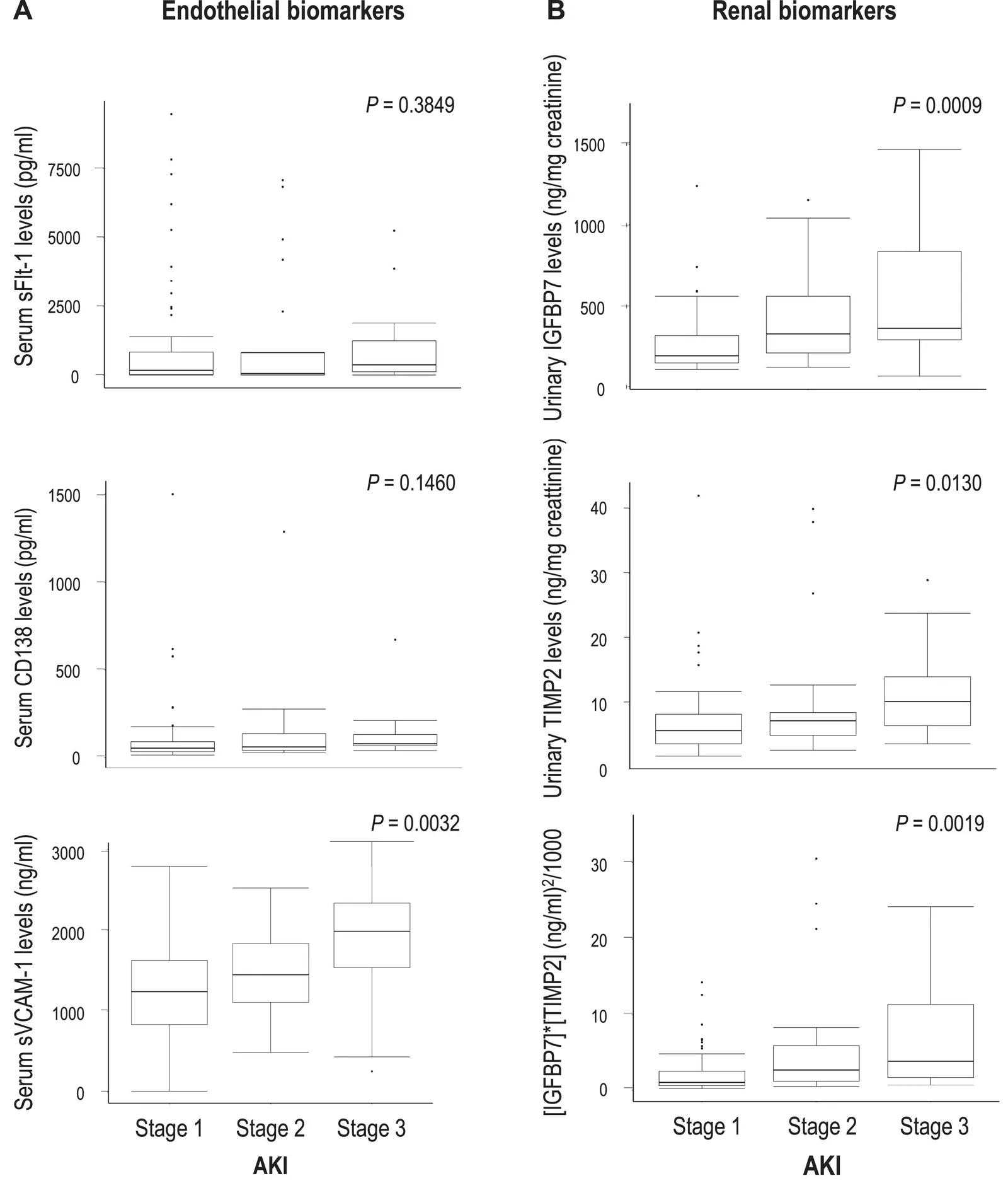
Serum endothelial (a) and urinary renal biomarker (b) values according to AKI stage at diagnosis. Data are median, interquartile range (boxes), non-outlier range (whiskers), and outliers (points). *P*-value derived from Kruskal–Wallis Test.

Markers of kidney stress—referred to here as renal biomarkers—and endothelial biomarkers showed different correlations with various aspects of AKI, including severity, duration, and outcomes, as shown in Table 3 and Fig. 2. At AKI diagnosis, urinary IGFBP7, TIMP2, and their product [IGFBP7]*[TIMP2] increased stepwise with AKI severity and correlated with kidney function markers, i.e. sCrea and eGFR, and changes in sCrea from baseline. In contrast, serum sFlt-1 and CD138 did not correlate with AKI stage, kidney function, or inflammation (as measured by CRP), but showed positive correlations with AKI duration and, for CD138, the length of hospitalization. Both biomarkers showed a negative correlation with follow-up eGFR. sVCAM-1 was the only endothelial marker linked to AKI severity but not to poor outcomes. Notably, sFlt-1 and CD138 were found to be correlated (rs = 0.466, *P *< 0.01), suggesting that they may be related in their pathways.

**Table 3:** Correlation analysis of biomarker levels and clinical data at AKI diagnosis and outcomes.

Spearman’s rank correlation coefficient						
	Endothelial biomarkers			Renal biomarkers		
	sFlt-1	CD138	sVCAM-1	IGFBP7	TIMP2	[IGFBP7]*[TIMP2]
***At AKI diagnosis***						
sCrea	0.110	0.221*	0.404**	0.289**	0.304**	0.315**
eGFR	0.036	−0.145	−0.388**	−0.240*	−0.305**	−0.281**
CRP	0.025	0.140	0.242*	0.192	0.216*	0.222*
Proteinuria	0.076	0.245*	0.303**	0.431**	0.597**	0.565**
sFlt-1		0.466**	−0.080	0.121	0.197	0.184
CD138			0.124	0.250*	0.349**	0.324**
sVCAM-1				0.248*	0.139	0.226*
IGFBP7					0.592**	0.881**
TIMP2						0.881**
***Outcomes***						
Change in sCrea^1^	0.060	0.196	0.368**	0.436**	0.358**	0.417**
Duration of AKI	0.321**	0.395**	0.128	0.170	0.159	0.181
Length of hospitalization^2^	0.187	0.243*	0.060	0.182	0.099	0.152
Follow-up eGFR^2^	−0.447**	−0.304**	−0.101	−0.065	−0.145	−0.110

^1^At AKI diagnosis to baseline. ^2^Outcomes assessed in survivors (*n* = 85) ≥90 days after AKI diagnosis; average follow-up time 5 ± 2 months. *P < 0.05; **P < 0.01. All urine (*n* = 89) and serum (*n* = 93) samples were collected at the time of AKI diagnosis. Abbreviations and units: Age, in years; Duration of AKI, in days; CD138, syndecan-1 (pg/ml); sCrea, serum creatinine (mg/dl); CRP, C-reactive protein (mg/dl); sFlt-1, soluble Fms-like tyrosine kinase-1 (pg/ml); eGFR, estimated glomerular filtration rate (ml/min/1.73 m^2^); IGFBP7; urinary insulin-like growth factor-binding protein 7 (ng/mg creatinine); [IGFBP7]*[TIMP-2] ((ng/ml)²/1 000); length of hospitalization, in days; proteinuria, mg protein/mg creatinine; TIMP2, urinary tissue inhibitor of metalloproteinase 2 (ng/mg creatinine); and sVCAM-1, serum soluble vascular cell adhesion molecule-1 (ng/ml).

**Figure 3:**
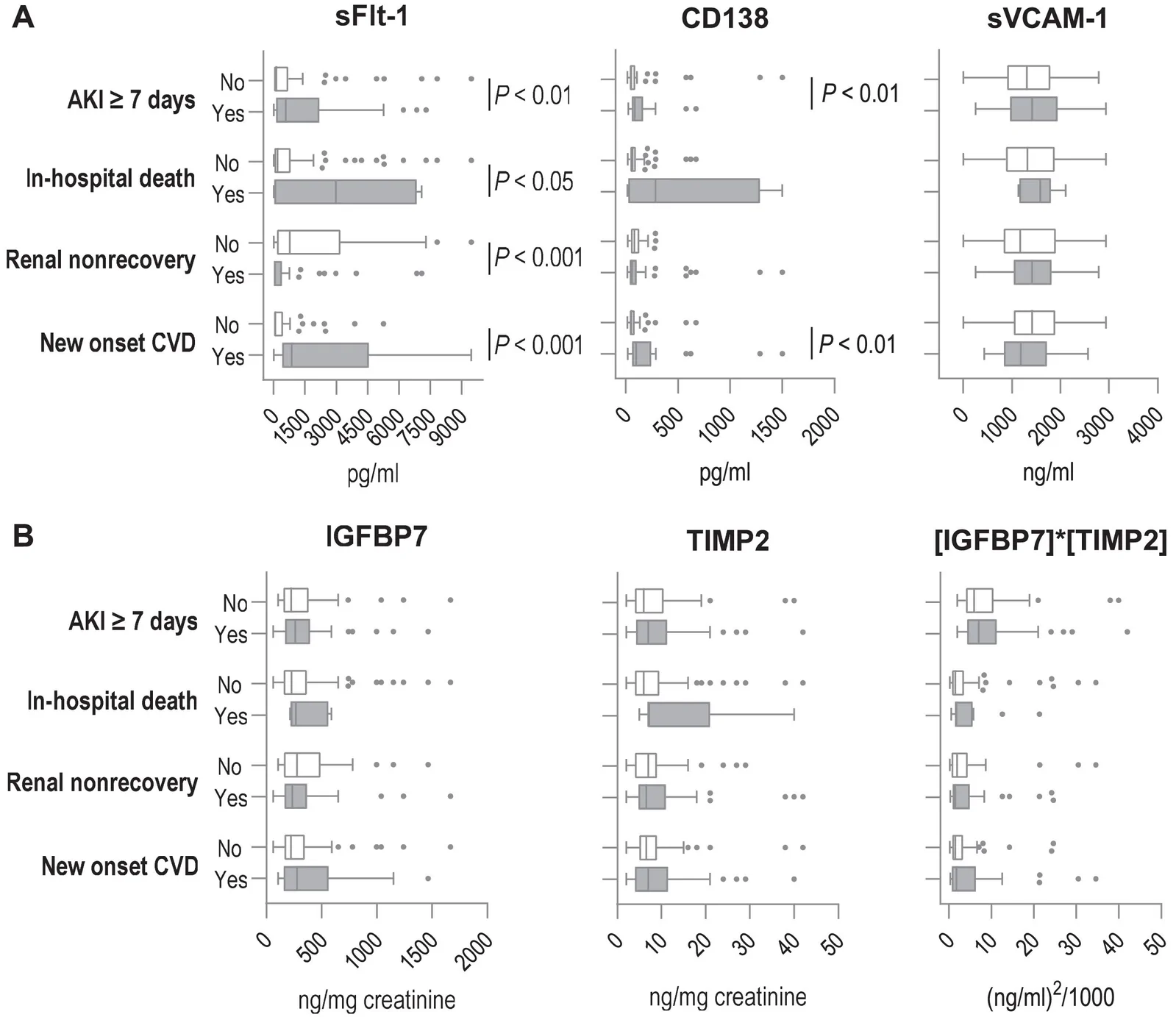
Univariable analysis of endothelial (a) and renal (b) biomarkers in relation to AKI outcomes. Boxplots show associations between biomarker levels and prolonged AKI duration, in-hospital mortality, incidence of new-onset CVD, and lack of renal recovery at ≥90 days post-AKI diagnosis. Data are presented as median (line), interquartile range (box), non-outlier range (whiskers), and individual outliers (dots). *P*-value derived from the Mann–Whitney U test.

As confirmed by univariable analysis (Fig. 3), these data suggest that endothelial biomarkers were more closely associated with poor outcomes, while renal biomarkers provide more insights into the initial injury severity.

For comparison purposes, baseline characteristics and outcomes were also stratified according to KDIGO stage (Supplementary Tables S3 and S4). As expected, urinary IGFBP7, TIMP-2, and their product increased stepwise with AKI severity, whereas sFlt-1 and CD138 were not associated with KDIGO stage. In contrast, sVCAM-1 increased with higher stages of AKI. Persistent AKI was more frequent in higher stages, and follow-up eGFR was lower in stage 3 AKI; however, renal nonrecovery and new-onset CVD did not differ significantly across KDIGO stages. No stage-dependent differences in mortality were observed, although the small number of events limits interpretation.

### Performance of endothelial and renal biomarkers in predicting outcomes

**Figure 4:**
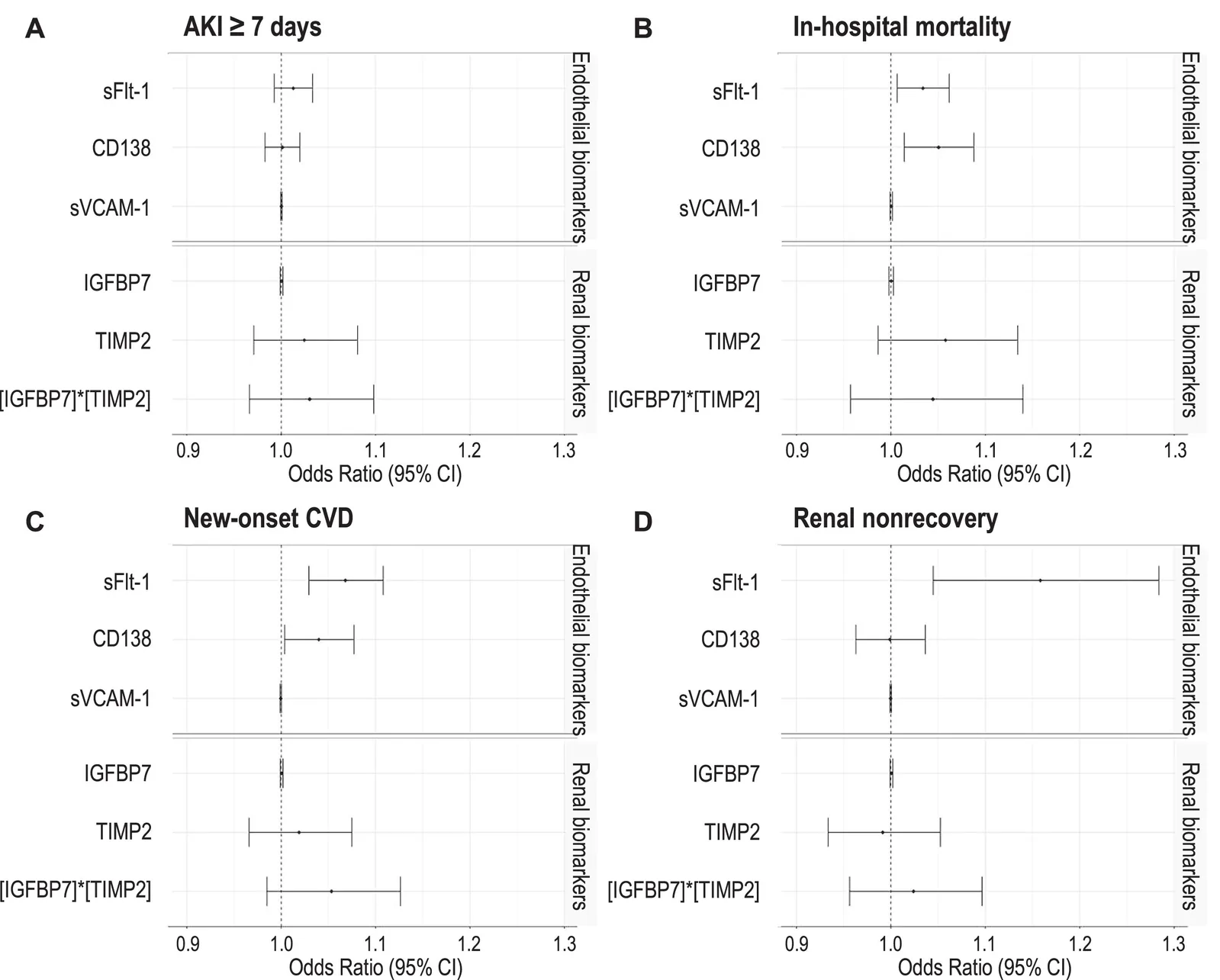
Association of endothelial and renal biomarkers with AKI outcomes: long AKI duration (a), in-hospital death (b), incidence of new-onset CVD (c), and renal nonrecovery at ≥90 days after diagnosis (d). Forest plots showing the OR and 95% CI associated with a 100-unit, 10-unit, and 1-unit increase in sFlt-1 (pg/ml), CD138 (pg/ml), and all other variable levels (sVCAM-1, ng/ml; IGFBP7, ng/mg creatinine; TIMP2, ng/mg creatinine; and [IGFBP7]*[TIMP-2] ((ng/ml)²/1000), respectively.

Among the endothelial biomarkers, serum sFlt-1 and CD138 levels were predictive of in-hospital mortality and the incidence of new-onset CVD, as shown in unadjusted analyses (Fig. 4). Each 100-unit increase in sFlt-1 is associated with a 3.4% increase in the odds of in-hospital mortality [odds ratio (OR) 1.034; 95% confidence interval (CI), 1.007–1.062; *P* = 0.0144] and a 6.8% increase in the odds of new-onset CVD (OR 1.068; 95% CI, 1.029–1.108; *P* = 0.0005). A 10-unit increase in CD138 is associated with a 5% increase in the odds of in-hospital mortality (OR 1.050; 95% CI, 1.014–1.088; *P* = 0.006) and a 4% increase in the odds of new-onset CVD (OR 1.040; 95% CI, 1.004–1.077; *P* = 0.0304). Additionally, serum sFlt-1 levels predicted renal nonrecovery, with a 15.8% increase in the odds of this outcome per 100-unit increase in sFlt-1 (OR 1.158; 95% CI, 1.045–1.284; *P* = 0.0052). In contrast, renal biomarkers did not demonstrate predictive value for any of the outcomes of interest. Interestingly, of the 2 anuric patients, only the patient with sFlt-1 levels in the highest tertile did not recover renal function at ≥90 days; the patient with low sFlt-1 levels recovered.

To further assess the utility of these biomarkers, we incorporated well-established clinical risk factors for AKI-related outcomes—such as age, sex, comorbidities, pre-existing renal dysfunction, and AKI severity—into a multivariable regression model. In this adjusted analysis, elevated sFlt-1 levels were independently associated with an increased risk of new-onset CVD and renal nonrecovery, whereas higher CD138 levels were independently associated with in-hospital mortality (Table 4). Moreover, the primary reason for hospitalization was not associated with sFlt-1 levels (Supplementary Table S2), nor with clinical outcomes (Supplementary Fig. S1).

**Table 4:** Adjusted association of endothelial biomarkers with AKI outcomes: long AKI duration, in-hospital death, incidence of new-onset CVD, and renal nonrecovery at ≥90 days after diagnosis.

	sFlt-1	CD138	sVCAM-1
AKI ≥ 7 days	1.024 (0.994–1.054)	1.011 (0.981–1.042)	1.000 (0.999–1.002)
In-hospital mortality	1.029 (0.987–1.074)	1.059 (1.003–1.118)*	1.001 (0.999–1.002)
New-onset CVD	1.145 (1.052–1.246)*	1.055 (0.990–1.126)	0.999 (0.998–1.000)
Renal nonrecovery	1.231 (1.050–1.444)**	0.962 (0.881–1.052)	1.000 (0.999–1.002)

Values expressed as adjusted OR (95% CI) per 100-unit, 10-unit, and 1-unit increase in sFlt-1 (pg/ml), CD138 (pg/ml), and sVCAM-1 (ng/ml), respectively, adjusted for clinical covariates (including age, sex, baseline estimated glomerular filtration rate, history of hypertension, diabetes mellitus, or CVD, change in sCrea from baseline to diagnosis, AKI stage, proteinuria, body mass index, and CRP) and endothelial and renal biomarkers. For in-hospital mortality, OR was adjusted for endothelial and renal biomarkers only. AKI, acute kidney injury; CD138, syndecan-1; and sFlt-1, soluble Fms-like tyrosine kinase-1. *P < 0.05, **P < 0.005.

**Figure 5:**
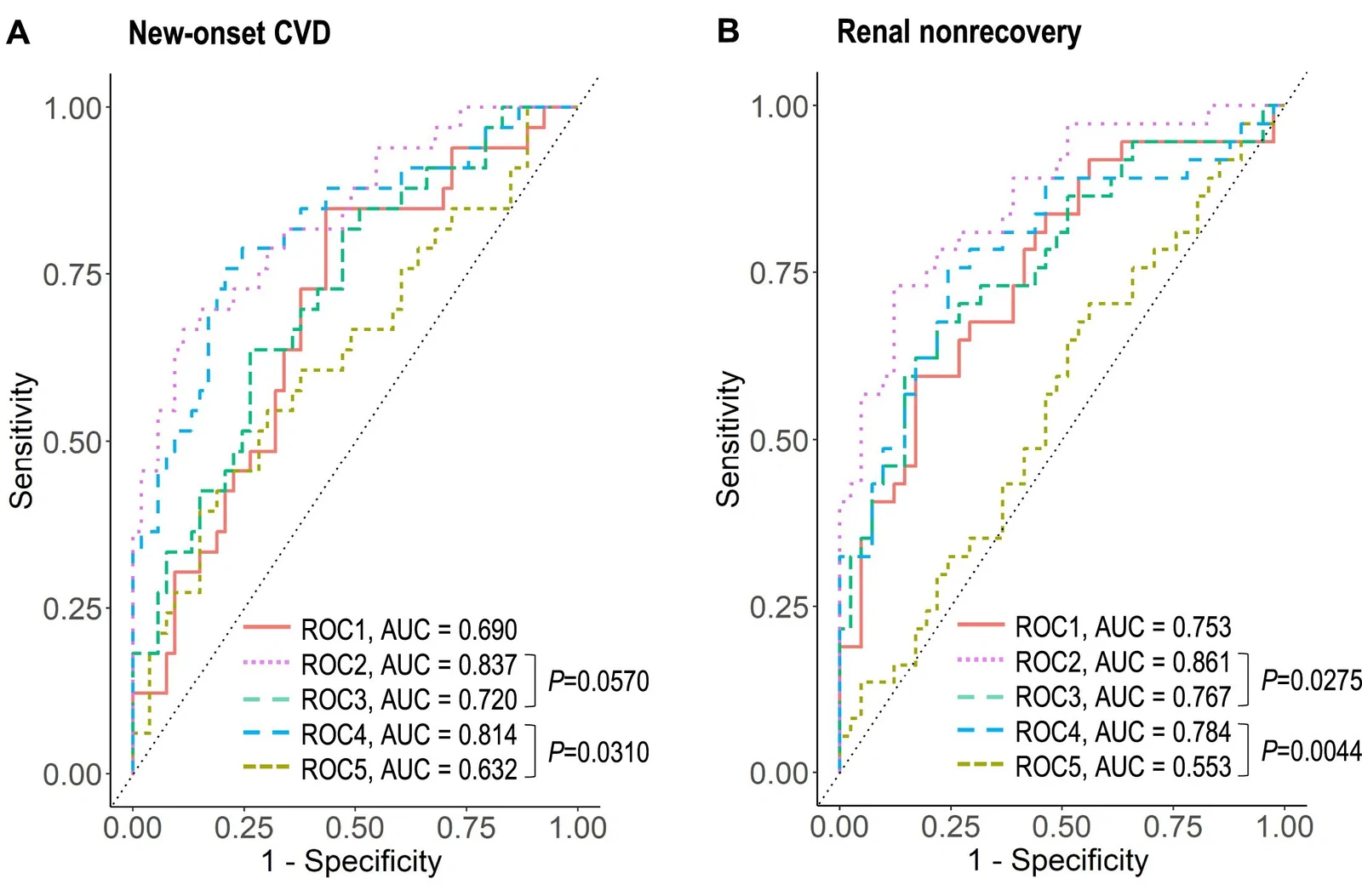
Receiver operating characteristic (ROC) curves showing the performance of the biomarker panels, both alone and in combination with the clinical variables, for the AKI outcomes: incidence of new-onset CVD (a), and renal nonrecovery at ≥90 days after diagnosis (b). The clinical model (ROC1) includes age, sex, baseline estimated glomerular filtration rate, history of hypertension, diabetes mellitus, or CVD, change in sCrea from baseline to diagnosis, AKI stage, proteinuria, body mass index, and CRP. ROC2 and ROC3 represent models with incremental additions: ROC2, clinical model combined with endothelial biomarkers, and ROC3, clinical model combined with renal biomarkers. ROC4 and ROC5 represent biomarker-only models: ROC4 includes endothelial biomarkers, and ROC5 includes renal biomarkers. *P*-values represent comparisons to the clinical model (ROC1). AKI, acute kidney injury; AUC, area under the curve.

ROC curves were used to evaluate the predictive performance of the biomarker panels, both alone and in combination with the clinical prediction model. The endothelial biomarkers demonstrated higher areas under the curve (AUCs) than the clinical model alone for predicting renal nonrecovery and new-onset CVD, although these differences were not statistically significant (Fig. 5 and Table 5). When endothelial markers were added to the clinical model, the AUC improved from 0.75 to 0.86 (95% CI, 0.64–0.86 and 0.78–0.94, respectively; *P* = 0.0163) for renal nonrecovery and from 0.69 to 0.84 for new-onset CVD (95% CI, 0.58–0.80 and 0.75–0.92, respectively; *P* = 0.0059). In contrast, the addition of renal stress biomarkers to the clinical model did not improve its performance for either outcome (AUC 0.77; 95% CI, 0.66–0.87; *P* = 0.3448; and AUC 0.72; 95% CI, 0.61–0.83; *P* = 0.4276, respectively). Direct comparisons between biomarker models showed higher AUC values for the endothelial biomarker panel alone than for the renal stress biomarker panel alone in predicting both renal nonrecovery (AUC 0.78; 95% CI, 0.68–0.89; vs. AUC 0.55; 95% CI, 0.42–0.68; *P* = 0.0044) and new-onset CVD (AUC 0.81; 95% CI, 0.72–0.91; vs. AUC 0.63; 95% CI, 0.51–0.76; *P* = 0.0310) (Fig. 5 and Table 5).

**Table 5:** Prognostic performance of the biomarker panels, both alone and in combination with the clinical prediction model, for the AKI outcomes: long AKI duration, in-hospital death, incidence of new-onset CVD, and renal nonrecovery at ≥90 days after diagnosis.

	AKI ≥ 7 days		In-hospital mortality		New-onset CVD		Renal nonrecovery	
	AUC (95% CI)	*P*-Value	AUC (95% CI)	*P*-Value	AUC (95% CI)	*P*-Value	AUC (95% CI)	*P*-Value
**Model: *Full model***								
	0.789 (0.691–0-888)	–	0.775 (0.505–1.000)	–	0.900 (0.828–0.971)	–	0.864 (0.783–0.994)	–
**ROC1: *Clinical model***								
	0.756 (0.648–0.864)	–	–	–	0.690 (0.575–0.805)	–	0.753 (0.644–0.863)	–
**ROC2: *Clinical model + endothelial biomarkers***								
	0.770 (0.668–0.873)	.6396	–	–	0.837* (0.748–0.925)	.0059	0.861** (0.780–0.942)	.0163
**ROC3: *Clinical model + renal biomarkers***								
	0.765 (0.658–0.871)	.6010	–	–	0.720 (0.610–0.830)	.4276	0.767 (0.661–0.874)	.3448
**ROC 4: *Endothelial biomarkers only***								
	0.601 (0.478–0.725)	.0695	0.751 (0.455–1.000)	.2605	0.814^#^ (0.717–0.911)	.0601	0.784^##^ (0.678–0.890)	.6996
**ROC 5: *Renal biomarkers only***								
	0.509 (0.373–0.645)	.0015	0.711 (0.546–0.876)	.6765	0.632 (0.508–0.757)	.4982	0.553 (0.424–0.682)	.0124

*P*-values represent comparisons to the clinical model (ROC1) or the full model for in-hospital mortality. *P = 0.0570 and **P = 0.0275 vs. ROC3; ^#^*P *= 0.0310 and ^##^*P *= 0.0044 vs. ROC5. Model (full model): includes clinical variables and endothelial and renal biomarker panels. For in-hospital mortality, the full model includes only the endothelial and renal biomarkers. ROC1 (clinical model): includes age, sex, baseline estimated glomerular filtration rate, history of hypertension, diabetes mellitus, or CVD, change in sCrea from baseline to diagnosis, AKI stage, proteinuria, body mass index, and CRP. ROC2 and ROC3: clinical model (ROC1) combined with endothelial biomarkers (ROC2) or renal biomarkers (ROC3), respectively. ROC4 and ROC5: biomarker-only models—ROC4 includes endothelial biomarkers, and ROC5 includes renal biomarkers. For in-hospital mortality, only the biomarker-only models (ROC4 and ROC5) and the full model were used. AKI, acute kidney injury; AUC, area under the curve; CI, confidence interval; and ROC, receiver operating characteristic.

Neither endothelial nor renal stress biomarkers significantly improved the AUC of the clinical model for the outcome of AKI duration. Notably, while the AUC of the endothelial biomarker panel alone (AUC 0.60; 95% CI, 0.48–0.73; *P* = 0.0695) did not differ significantly from that of the clinical model (AUC 0.76; 95% CI, 0.65–0.86), the AUC for the renal stress biomarker panel alone was significantly lower (AUC 0.51; 95% CI, 0.37–0.65; *P* = 0.0015). Due to the low number of events, evaluating the combination of the full biomarker panel with the clinical model for in-hospital mortality was not feasible. Nonetheless, among the biomarker-only models, the combined panel yielded the highest AUC (0.78; 95% CI, 0.51–1.00), followed by the endothelial panel (AUC 0.75; 95% CI, 0.46–1.00) and the renal stress panel (AUC 0.71; 95% CI, 0.55–0.88). AUC values and corresponding statistics are summarized in Table 5.

To account for potential heterogeneity of AKI pathophysiology, we performed additional multivariable logistic regression analyses for both main outcomes, incorporating the three major AKI mechanisms—postoperative, nephrotoxic, and perfusion-related AKI—as covariates, together with age, sex, pre-existing renal dysfunction, and AKI severity. Because AKI etiologic categories were represented by relatively small numbers of patients, these analyses were based on a parsimonious adjustment model than multivariable models shown in Table 4. After adjustment for AKI etiology, sFlt-1 remained independently associated with both new-onset CVD (Table 6) and renal nonrecovery (Table 7, Supplementary Table S5). In this reduced model, CD138 was also associated with new-onset CVD even after adjustment for AKI mechanism (Table 6), whereas sVCAM-1 was not associated with either outcome. In ROC analyses, inclusion of the major AKI causes in models containing the respective endothelial marker and clinical covariates did not materially affect discrimination for either outcome (Tables 6 and 7). Notably, models including sFlt-1 showed discriminatory performance comparable to that of the full model and superior to that of the corresponding models including sVCAM-1 for new-onset CVD and either CD138 or sVCAM-1 for renal nonrecovery.

**Table 6:** Association of endothelial biomarkers with incidence of new-onset CVD after adjustment for major AKI mechanisms and incremental predictive value beyond AKI etiology.

Model/Predictor	OR (95% CI)	*P*-Value	AUC (95% CI)	*P-Value
**Model: *Full model***				
• sFlt-1	1.089 (1.034–1.148)	.0014	0.8409 (0.7531–0.9287)	–
• CD138	1.014 (0.982–1.047)	.4008	0.8409 (0.7531–0.9287)	–
• sVCAM-1	0.999 (0.998–1.000)	.2227	0.8409 (0.7531–0.9287)	–
**Model 1: *Each* e*ndothelial biomarker separately + clinical covariates***				
• sFlt-1	1.072 (1.030–1.115)	.0006	0.7843 (0.6830–0.8857)	–
• CD138	1.037 (1.001–1.075)	.0429	0.7141** (0.6000–0.8283)	–
• sVCAM-1	0.999 (0.999–1.000)	.1741	0.6763**^,#^ (0.5631–0.7894)	–
**Model 2: *Model 1 + perfusion-related AKI***				
• sFlt-1	1.072 (1.030–1.115)	0.0006	0.7864 (0.6843–0.8884)	.7829
• CD138	1.037 (1.001–1.075)	.0417	0.7202** (0.6066–0.8338)	.2935
• sVCAM-1	0.999 (0.999–1.000)	.1595	0.6717**^,##^ (0.5582–0.7853)	.6567
**Model 3: *Model 1 + nephrotoxic AKI***				
• sFlt-1	1.077 (1.034–1.122)	.0003	0.8061 (0.7080–0.9041)	.4517
• CD138	1.038 (1.001–1.077)	.0427	0.7278** (0.6157–0.8399)	.5825
• sVCAM-1	.999 (0.999–1.000)	.1810	0.6773**^,##^ (0.5638–0.7908)	.9590
**Model 4: *Model 1 + postoperative AKI***				
• sFlt-1	1.080 (1.035–1.127)	.0004	0.8146 (0.7176–0.9117)	.3699
• CD138	1.036 (1.002–1.072)	.0376	0.7449** (0.6375–0.8524)	.3958
• sVCAM-1	0.999 (0.999–1.000)	.1326	0.7010**^,##^ (0.5871–0.8149)	.5156

*P-values represent comparisons with the Model 1 containing the same endothelial marker. **P < 0.05 vs. the full model; ^#^*P *= 0.0542 and ^##^*P *< 0.05 vs. the model including sFlt-1. Values are expressed as adjusted ORs (95% CI) per 100-unit, 10-unit, and 1-unit increase in sFlt-1 (pg/ml), CD138 (pg/ml), and sVCAM-1 (ng/ml), respectively. The OR reflects the strength of association for an individual predictor variable (e.g. sFlt-1, CD138 or sVCAM-1), whereas the area under the receiver operating characteristic curve (AUC) reflects the overall discriminatory performance of the model. The full model included all three endothelial biomarkers, the major AKI mechanisms, and the clinical covariates age, sex, baseline estimated glomerular filtration rate, and AKI stage. Model 1 included each endothelial biomarker separately in combination with the clinical covariates. Models 2, 3, and 4 comprised Model 1 combined with perfusion-related AKI, nephrotoxic AKI or postoperative AKI, respectively. AKI, acute kidney injury; CD138, syndecan-1; CI, confidence interval; sFlt-1, soluble Fms-like tyrosine kinase-1; and sVCAM-1, soluble vascular cell adhesion molecule-1.

**Table 7:** Association of endothelial biomarkers with renal nonrecovery at ≥90 days after AKI diagnosis after adjustment for major AKI mechanisms and incremental predictive value beyond AKI etiology.

Model/Predictor	OR (95% CI)	*P*-Value	AUC (95% CI)	*P-Value
**Model: *Full model***				
• sFlt-1	1.212 (1.060–1.386)	.0048	0.8511 (0.7721–0.9301)	–
• CD138	0. 949 (0.882–1.022)	.1650	0.8511 (0.7721–0.9301)	–
• sVCAM-1	1.000 (0.999–1.001)	.7269	0.8511 (0.7721–0.9301)	–
**Model 1: *Each* e*ndothelial biomarkerseparately + clinical covariates***				
• sFlt-1	1.184 (1.056–1.328)	.0039	0.8461 (0.7633–0.9289)	–
• CD138	.997 (0.957–1.037)	.8650	0.6889**^,#^ (0.5761–0.8017)	–
• sVCAM-1	1.000 (0.999–1.000)	.3652	0.6878**^,#^ (0.5757–0.7999)	–
**Model 2: *Model 1 + perfusion-related AKI***				
• sFlt-1	1.184 (1.056–1.328)	.0039	0.8478 (0.7655–0.9301)	.3381
• CD138	0.996 (0.957–1.037)	.8526	0.6944**^,#^ (0.5815–0.8074)	.5814
• sVCAM-1	1.000 (0.999–1.000)	.3928	0.6894**^,#^ (0.5776–0.8012)	.6798
**Model 3: *Model 1 + nephrotoxic AKI***				
• sFlt-1	1.178 (1.048–1.324)	.0060	0.8500 (0.7691–0.9309)	.7219
• CD138	0.996 (0.956–1.036)	.8296	0.7194**^,#^ (0.6099–0.8290)	.3307
• sVCAM-1	1.000 (0.999–1.000)	.3087	0.7317**^,#^ (0.6247–0.8386)	.1502
**Model 4: *Model 1 + postoperative AKI***				
• sFlt-1	1.187 (1.057–1.334)	0.0039	0.8439 (0.7604–0.9274)	.7128
• CD138	0.997 (0.957–1.038)	.8687	0.6889**^,#^ (0.5731–0.8017)	1.0000
• sVCAM-1	1.000 (0.999–1.000)	.3660	0.6883**^,#^ (0.5763–0.8003)	.7541

*P-values represent comparisons with the Model 1 containing the same endothelial marker. **P < 0.01 vs. the full model; ^#^*P *= 0.01 vs. the corresponding model including sFlt-1. Values are expressed as adjusted odds ratios (95% CI) per 100-unit, 10-unit, and 1-unit increase in sFlt-1 (pg/ml), CD138 (pg/ml), and sVCAM-1 (ng/ml), respectively. The OR reflects the strength of association for an individual predictor variable (e.g. sFlt-1, CD138, or sVCAM-1), whereas the area under the receiver operating characteristic curve (AUC) reflects the overall discriminatory performance of the model. The full model included all three endothelial biomarkers, the major AKI mechanisms, and the clinical covariates age, sex, baseline estimated glomerular filtration rate, and AKI stage. Model 1 included each endothelial biomarker separately in combination with the clinical covariates. Models 2, 3, and 4 comprised Model 1 combined with perfusion-related AKI, nephrotoxic AKI or postoperative AKI, respectively. AKI, acute kidney injury; CD138, syndecan-1; CI, confidence interval; sFlt-1, soluble Fms-like tyrosine kinase-1; and sVCAM-1, soluble vascular cell adhesion molecule-1.

## DISCUSSION

We hypothesized that endothelial biomarkers—reflecting endothelial dysfunction or injury—predict adverse outcomes following AKI. Indeed, in this longitudinal study of unselected, hospitalized non-critically ill patients who developed AKI during their hospital stay, we observed that endothelial biomarkers—beyond biomarkers of kidney stress—provided complementary prognostic information for renal nonrecovery and poor outcomes, including in-hospital mortality and new-onset CVD, even in a cohort predominantly affected by mild AKI.

AKI is a common and serious complication among hospitalized patients, resulting from a wide range of insults and underlying conditions [9]. Given its multifactorial nature, understanding the biological processes, the severity of kidney damage, and recovery potential is clinically vital. Although traditional clinical parameters are of great importance, biomarkers can offer deeper insight into AKI risk stratification and enable the identification of subphenotypes linked to distinct molecular pathways and adverse clinical outcomes.

Among the key drivers of AKI progression is tubular epithelial injury, which can promote inflammation, fibrosis, and maladaptive repair—hallmarks of chronic renal dysfunction [17, 18]. Cell cycle arrest at the G2/M phase is one such mechanism contributing to the release of profibrotic cytokines [28]. Urinary [IGFBP7]*[ TIMP2] has been implicated in cell cycle arrest and AKI onset prediction [12–14] but also in the prediction of renal nonrecovery when measured on the first day of AKI diagnosis [10, 15, 16]. In our study population, the renal biomarkers IGFBP7 and TIMP2—as well as their combined product [IGFBP7]*[TIMP2]—reflected the severity of AKI as defined by the KDIGO guidelines but did not provide additional prognostic value, even when integrated with clinical models. Notably, although clinical studies have demonstrated that [IGFBP7]*[TIMP2] is a robust tool for early AKI prediction, its prognostic value after AKI diagnosis—particularly in less acutely ill or elective populations such as ours—remains uncertain. In addition, missing values in two anuric patients and the timing of urinary stress marker measurements may have introduced disadvantages for these biomarkers, potentially limiting their predictive performance [15, 29–31].

Elevated circulating endothelial markers, such as sFlt-1 and CD138, indicate a systemic disease state that can affect multiple vascular beds. Impaired endothelial function contributes to the pathobiology of AKI in many ways [28, 32]. Furthermore, inflammation and oxidative stress generated during AKI can disrupt endothelial integrity, leading to endothelial activation, degradation of the eGC, and vascular dysfunction within the kidney and in distant organs, including the heart [32–35]. Importantly, endothelial markers measured at the time of AKI cannot discriminate between pre-existing endothelial dysfunction and AKI-induced endothelial injury; nonetheless, their high levels may reflect increased vulnerability to further vascular insults and a reduced capacity to coordinate injury responses and support (renal) repair during this critical phase [36–39]. Moreover, endothelial dysfunction, together with inflammation, appears to constitute a shared mechanistic pathway mediating the crosstalk between renal and cardiac dysfunction [40, 41].

Circulating sFlt-1 levels are elevated in various diseases with renal impairment [42]. In the TRIBE-AKI cohort of 1444 patients, Mansour *et al*. observed increased sFlt-1 levels after cardiac surgery, particularly in those with adverse outcomes such as prolonged AKI duration and all-cause mortality [8]. Similarly, two independent kidney transplant cohorts found that high sFlt-1 levels within the first week post-transplant were associated with delayed graft function—a manifestation of AKI in these patients—as well as with impaired graft function at 1 year and increased all-cause mortality [37, 43]. Notably, sFlt-1 levels correlate negatively with peritubular capillary surface area, supporting its use as a marker of microvascular injury and capillary rarefaction in the kidneys [37]. Moreover, elevated sFlt-1 has been linked to cardiovascular events in patients with renal impairment or heart failure. While this association was not always independent [42, 44–46], it suggests a potential role for sFlt-1 in cardiorenal interactions.

CD138 is a proteoglycan that anchors heparan sulfate on the cell surface and plays a key role in inflammation and vascular permeability [47]. It is strongly associated with poor outcomes in AKI, particularly in the context of sepsis and cardiac surgery. Elevated CD138 levels have been shown to predict AKI progression after cardiac surgery and are linked to a higher risk of requiring kidney replacement therapy following surgery or sepsis [48–50]. It is a critical biomarker for endothelial damage—a shared mechanism in AKI and CVD pathogenesis [51]. Elevated CD138 may identify patients at risk for systemic vascular dysfunction, warranting closer CVD monitoring.

Notably, sVCAM-1, a systemic biomarker reflecting potential endothelial cell dysfunction, showed a stepwise increase with AKI severity in this cohort. However, it lacked any association with changes in serum levels of sFlt-1 and CD138 and predictive power for adverse outcomes. These molecules are physiologically distinct, and the conditions under which one may be shed and released into circulation may differ, highlighting the mechanistic specificity of sFlt-1 and CD138 in our patient population [52, 53]. sFlt-1 binds electrostatically to proteoglycans on the eGC, and its levels have been associated with eGC damage in various disease settings [19, 21, 54]. Herein, we observed a strong correlation between sFlt-1 and CD138 levels, suggesting that eGC degradation may drive elevations in these biomarkers.

Given the heterogeneity of AKI, selecting the appropriate biomarker panels to identify clinically relevant subphenotypes remains a major challenge. Emerging strategies, including machine learning approaches and multiplex biomarker profiling, present promising avenues for addressing this complexity [55]. For instance, elevated levels of various endothelial biomarkers have been independently associated with increased risk of adverse outcomes following AKI, regardless of the underlying etiology [8, 9, 11, 37, 43, 53]. Further support for the role of endothelial dysfunction in AKI pathogenesis comes from preclinical studies, which demonstrate that interventions aimed at preserving or restoring endothelial integrity can improve outcomes [36, 37, 39, 56–58].

Our findings underscore the prognostic relevance of endothelial biomarkers, particularly sFlt-1 and CD138, concerning in-hospital mortality and the incidence of new-onset CVD, and sFlt-1 in predicting renal nonrecovery among hospitalized patients with AKI, independent of KDIGO stage. In contrast, sVCAM-1 was associated with AKI severity but not with outcome endpoints, suggesting distinct pathophysiological roles for these endothelial biomarkers. Because the KDIGO stage was included as a covariate in all adjusted analyses and higher-stage AKI was relatively infrequent in our cohort, additional subgroup analyses stratified by severity would have provided limited statistical power.

However, the predominance of KDIGO stage 1 AKI in our cohort, together with the high proportion of multifactorial AKI, is consistent with the epidemiology of AKI in non-critically ill hospitalized populations. Importantly, even mild AKI is associated with adverse renal and cardiovascular outcomes [1–5]. In this setting, biomarkers may be particularly valuable for identifying patients at risk of persistent injury or long-term complications when creatinine-based staging provides limited prognostic discrimination.

Despite the heterogeneous and frequently multifactorial nature of AKI in our cohort, adjustment for the major AKI mechanisms did not materially alter the association of endothelial biomarkers, particularly sFlt-1, with new-onset CVD or renal nonrecovery. This suggests that the prognostic value of sFlt-1 is not solely explained by differences in AKI etiology, but may reflect a broader endothelial injury signal across distinct AKI contexts. However, these analyses were based on a parsimonious adjustment model because of the limited number of patients in individual etiologic categories and should therefore be interpreted cautiously.

This study has several limitations that warrant consideration. Previous studies have demonstrated the value of these biomarkers in selected groups, such as cardiac surgery or transplant patients; our study extends this to a broader hospitalized cohort. Nevertheless, the study population consisted predominantly of non-critically ill inpatients, which limits the generalizability of our findings, particularly to patients with sepsis or those requiring intensive care, who represent the largest proportion of AKI cases in hospitalized settings. While this design limits external validity to critically ill populations, it reduces confounding related to sepsis-associated multiorgan dysfunction and enables focused evaluation of endothelial injury in non-critically ill hospitalized patients, an understudied but clinically relevant subgroup of AKI.

Further limitations include the modest sample size, which reduces statistical power for stratified analyses and outcome prediction, particularly for less frequent endpoints such as mortality. Mortality analyses should therefore be interpreted as exploratory. Reliance solely on sCre changes may have resulted in underdiagnosis of AKI cases, misclassification of AKI severity, and potential bias in estimating AKI duration. In addition, the exclusion of two anuric patients may have limited the prognostic performance of urinary renal stress biomarkers. Biomarker measurements were obtained at a single-time-point after confirmation of fulfillment of KDIGO criteria by a nephrologist, which precludes evaluation of pre-AKI levels or longitudinal changes. Although longitudinal measurements were not available, biomarker assessment at the time of AKI diagnosis may better reflect active pathological processes relevant to risk stratification [36].

Regarding renal recovery, as post-AKI proteinuria was not evaluated, an important marker may have been missed and, consequently, the results should be interpreted with caution [59]. Furthermore, it was assessed using follow-up eGFR measurements obtained ≥90 days after AKI; however, the timing of follow-up ranged from 3 to 7 months, which may have introduced heterogeneity in recovery assessment across patients. Finally, given the exploratory, hypothesis-generating design of the study, our results should be interpreted as preliminary signals rather than causal relationships and definitive evidence. These observations highlight potential mechanisms that warrant validation in future studies with dedicated experimental designs.

Ultimately, endothelial biomarkers offer valuable insights into vascular health and systemic inflammation, which are closely tied to renal outcomes. Their integration into clinical practice may help identify high-risk AKI subphenotypes that are not necessarily defined by the presence or absence of kidney epithelial injury, and guide more personalized follow-up or therapeutic strategies. Focusing on endothelial health in these patients could support more effective and individualized management.

## Supplementary Material

sfag277_Supplemental_Files

## Data Availability

Data are available upon request.
