# Supplementary material for: Endothelial biomarkers predict kidney and cardiovascular outcomes after acute kidney injury
Source: Clin Kidney J. 2026 Aug 13;19(9):sfag277. doi: 10.1093/ckj/sfag277 (PMC13542715; doi:10.1093/ckj/sfag277)
Supplement: Supplementary file 1 [file sfag277_supplemental_files.zip › Supplementary Figure.pdf]

Supplementary Figure

Vascular signals in AKI: endothelial biomarkers predict renal and cardiovascular outcomes after injury

Running head: Endothelial biomarkers as vascular signals in AKI

Giovana S. Di Marco[1], Katrin Beul[1]; Eike Bormann[2], Hermann Pavenstädt[1], Marcus Brand[1]

[1]Department of Internal Medicine D, University Hospital Muenster, Münster, Germany.

[2]Institute for Biostatistics and Clinical Research, University Hospital Münster, Muenster, Germany.

Corresponding author: Giovana S. Di Marco, Albert-Schweitzer-Campus 1, Gebäude A14, 48149 Münster, Germany. Phone: +49 251 8356911; E-mail: giodimarco@gmail.com; https://orcid.org/0000-0002-1877-638X

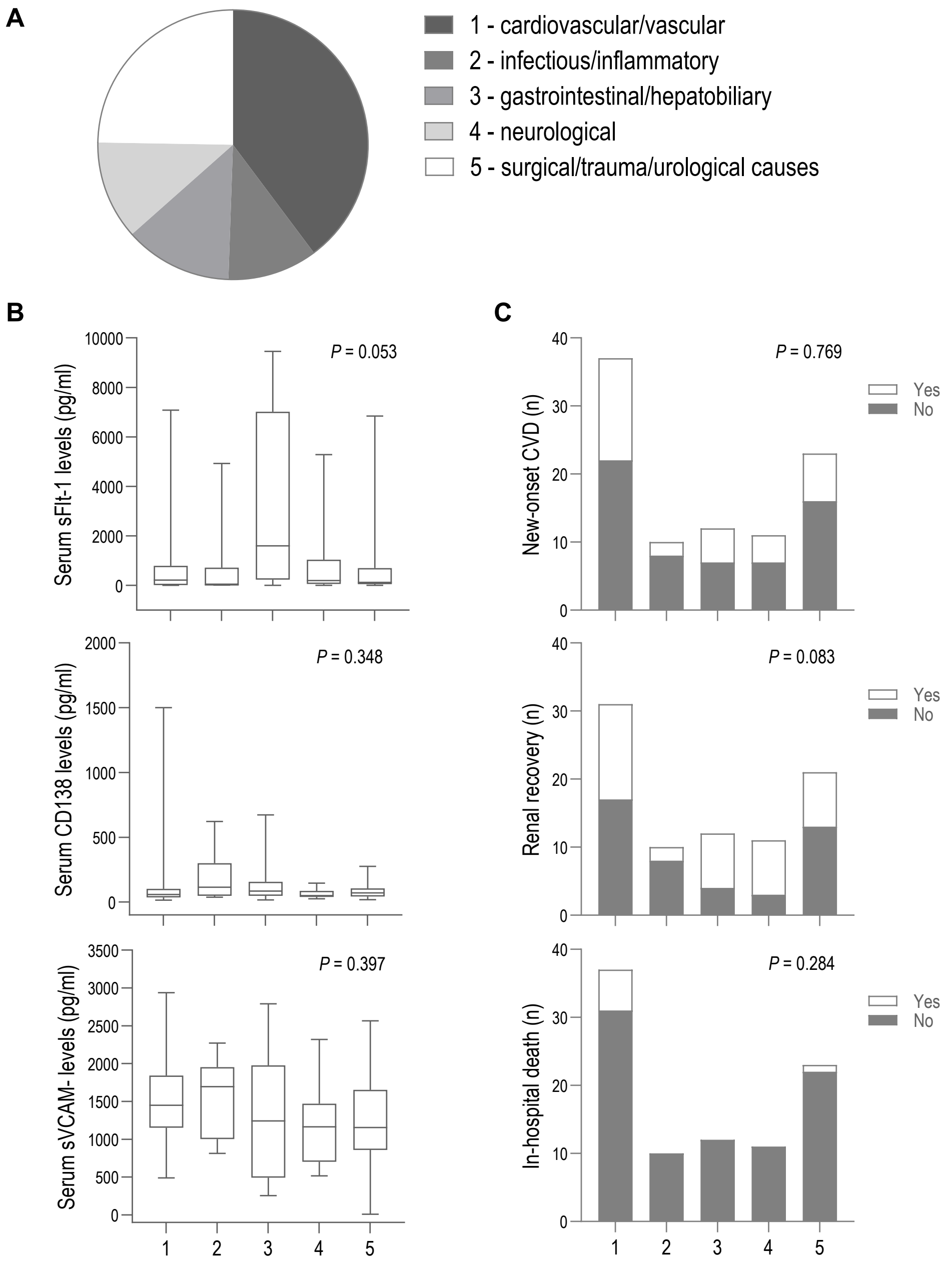

Figure S1. Overview of endothelial biomarkers and AKI outcomes stratified by reason for hospitalization. (A) The pie chart illustrates the proportional distribution of patients according to the primary reason for hospital admission, classified into five major clinical groups based on ICD-10 blocks: (1) cardiovascular/vascular (I10–I79, I20–I52), (2) infectious/inflammatory (A00–B99, J00–J22), (3) gastrointestinal/hepatobiliary (K20–K92), (4) neurological (I60–I69, G40–G99), and (5) surgical, trauma, or urological causes (S00–S99, N00–N39, T80–T88, Z96). (B) Box-and-whisker plots summarize the distribution of endothelial biomarker levels across hospitalization categories. Data are presented as median (line), interquartile range (box), and full date range (whiskers). P-value derived from the Kruskal-Wallis test. (C) Stacked bar charts show the relative distribution of AKI outcomes within each hospitalization group. P-value derived from the Fisher’s exact test.
