# Supplementary material for: Endothelial biomarkers predict kidney and cardiovascular outcomes after acute kidney injury
Source: Clin Kidney J. 2026 Aug 13;19(9):sfag277. doi: 10.1093/ckj/sfag277 (PMC13542715; doi:10.1093/ckj/sfag277)
Supplement: Supplementary file 1 [file sfag277_supplemental_files.zip › Supplementary methods.pdf]

Supplementary Methods

Vascular signals in AKI: endothelial biomarkers predict renal and cardiovascular outcomes after injury

Running head: Endothelial biomarkers as vascular signals in AKI

Giovana S. Di Marco[1], Katrin Beul[1]; Eike Bormann[2], Hermann Pavenstädt[1], Marcus Brand[1]

[1]Department of Internal Medicine D, University Hospital Muenster, Münster, Germany.

[2]Institute for Biostatistics and Clinical Research, University Hospital Münster, Muenster, Germany.

Corresponding author: Giovana S. Di Marco, Albert-Schweitzer-Campus 1, Gebäude A14, 48149 Münster, Germany. Phone: +49 251 8356911; E-mail: giodimarco@gmail.com; https://orcid.org/0000-0002-1877-638X

Methods (extended)

Settings and study population

This longitudinal study included 93 consecutive adult patients diagnosed with AKI and treated at the University Hospital Münster between August 2021 and July 2023. Ethical approval was obtained from the Joint Ethics Committee of the Landesärztekammer Westfalen-Lippe and the University of Münster (No. 2021-470-f-S), and all patients provided written informed consent.

Patients with significant changes in serum creatinine were identified via the hospital's electronic alert system, which automatically detects elevated serum creatinine (sCrea) levels and informs the attending nephrologist. Patients were evaluated by a nephrologist at the earliest possible opportunity (typically within four hours). At AKI diagnosis, here defined as the first time point at which KDIGO criteria fulfillment was confirmed by a nephrologist, urine and blood samples were collected immediately. AKI was defined according to KDIGO criteria: sCrea increase ≥0.3 mg/dL within 48 h or ≥1.5-fold from baseline within 7 days. Baseline sCrea was defined at the admission or the closest outpatient value. AKI stage was assigned per KDIGO: AKI Stage 1 (sCrea: 1.5 to 1.9-fold increase within 7 days or ≥ 0.3 mg/dl within 48h; or urine excretion: <0.5 ml/Kg/h over a period >6h); AKI Stage 2 (sCrea: 2- to 2.9-fold increase; or urine excretion: <0.5 ml/Kg/h for >12h) and AKI Stage 3 (sCrea: ≥ 3-fold increase or levels ≥ 4 mg/dl with an acute increase ≥ 0.5 mg/dl; or urine excretion: <0.3 ml/Kg/h for >24h or anuria >12h).

Exclusion criteria: age <18 years, missing consent, sepsis, admission to the intensive care unit, SARS-CoV-2 infection, stage 5 CKD/dialysis, organ transplantation, malignancy, pregnancy, or breastfeeding. Critically ill patients were excluded in order to minimize confounding, as this population is highly heterogeneous and characterized by severe, often multifactorial illness accompanied by pronounced

systemic inflammation. These conditions are known to substantially affect endothelial biomarker concentrations—particularly markers such as sFlt-1 and CD138—independently of AKI-specific pathophysiological mechanisms.[1–5] In addition, four patients had missing urine samples, including two who were anuric due to AKI stage 3. These patients were excluded from urine-based analyses but retained in all other analyses.

Follow-up and outcomes

Primary outcomes were AKI duration, in-hospital mortality, new-onset CVD, and renal nonrecovery ≥90 days post-AKI.

AKI duration was defined as the number of hospitalization days during which KDIGO serum creatinine criteria for AKI (increase ≥0.3 mg/dL within 48 hours or ≥1.5× baseline) were fulfilled. The start time was defined as the first time point at which KDIGO criteria were met ("time of diagnosis"), and AKI was considered resolved when the patient no longer fulfilled the KDIGO criteria. AKI duration was further categorized as short (<7 days) or persistent (≥7 days), in accordance with prior literature.[6]

New-onset CVD was defined as a physician-diagnosed, first occurrence of any cardiovascular condition during the follow-up period in patients without a prior history of that specific condition. Cardiovascular conditions included ischemic heart disease (single- and multi-vessel coronary artery disease and acute myocardial infarction), heart failure and cardiac decompensation, valvular heart disease with decompensation, cerebrovascular disorders (ischemic stroke and other cerebral infarctions), and peripheral and extracranial vascular disease (peripheral arterial disease and carotid artery stenting). Other cardiac disorders and cardiovascular-related presentations included cardiogenic shock, cardiac arrest, and hypertensive crisis.

Renal recovery was defined as an estimated eGFR value within at least 90% of the baseline eGFR as previously described.[7] This definition is based solely on eGFR trajectories and does not incorporate post-AKI proteinuria.

The follow-up period ranged from 3 to 7 months (≥ 90 days) after the AKI diagnosis to allow sufficient time for recovery. A 90-day time frame for recovery was used in line with Kidney Disease Outcomes Quality Initiative guidelines defining chronic kidney disease as kidney dysfunction lasting >90 days.[8] Data were taken from the nearest available clinical visit occurring at least 90 days after diagnosis. For patients who did not return for hospital follow-up visits, retrospective data were collected by contacting the patient's family physician.

Data collection and laboratory analyses

Detailed demographic, laboratory, and clinical data were obtained from a computerized database. Routine laboratory exams and biochemical tests were performed in the Centre for Laboratory Medicine. All GFR values were estimated using the CKD-EPI equation.

Blood and urine samples (without stabilizers) were collected at enrollment for biomarker testing. After centrifugation (2 min at 5,000g for urine; 10 min at 2,000g for serum), samples were aliquoted in clean polypropylene tubes, frozen, and stored at -80°C.

Serum sFlt-1 (human sVEGF R1/Flt-1, DVR100B, R&D Systems, USA), CD138 (syndecan-1, 950.640, Diaclone, France), and sVCAM-1 (human VCAM-1/CD106, DY809, R&D Systems, USA), as well as urinary IGFBP7 (human IGFBP-rp1/IGFBP7, DY1334, R&D Systems, USA) and TIMP2 (human TIMP2, DY971, R&D Systems, USA), were measured using commercial ELISA kits. Assays were performed in duplicate according to the manufacturers' instructions. For sVCAM-1, IGFBP7, and

TIMP2, samples were diluted 2000-, 50-, and 500-fold, respectively; values exceeding the upper assay limit were remeasured after 10-fold dilution. Urinary biomarkers were normalized to creatinine. The [IGFBP7]*[TIMP2] product was calculated by multiplying IGFBP7 and TIMP2 concentrations (ng/mL) and dividing by 1000.[9]

Statistical analysis

Categorial variables are presented as absolute and relative frequencies. Continuous variables are presented as mean and standard deviation for normally distributed variables, or as median with 25th and 75th percentiles for non-normally distributed variables. Fisher's exact test was used to compare categorical variables between groups. ANOVA was used to compare normally distributed continuous variables across three groups, and the Kruskal–Wallis test was used for non-normally distributed continuous variables. Spearman's rank correlation coefficient was used to evaluate the correlation among the biomarkers sFlt-1, CD138, sVCAM-1, IGFBP7, TIMP2, and [IGFBP7]*[TIMP2], as well as between the biomarkers and the variables: sCrea, baseline eGFR, C-reactive protein (CRP), proteinuria, change in sCrea, duration of AKI, length of hospitalization, and follow-up eGFR.

Continuous biomarkers were modeled in increments that were clinically meaningful (100 units for sFlt-1 and 10 units for CD138), based on their observed distributions, to enhance the interpretability of regression estimates. Tertiles of sFlt-1 were used exclusively for descriptive analyses, and stratification by tertiles was performed for sFlt-1, the biomarker with the strongest association with outcome variables in preliminary analyses. KDIGO stage was included as a covariate in all adjusted models to account for AKI severity. Univariate logistic regression was used to evaluate the influence of sFlt-1, CD138, and sVCAM-1 on AKI duration $\geq$ 7 days, in-hospital mortality, new-onset CVD, and renal nonrecovery. Additionally, a multivariable

logistic regression model was used to evaluate the effects of sFlt-1, CD138, and sVCAM-1 on AKI duration ≥ 7 days, new-onset CVD and renal nonrecovery, adjusting for age, sex, baseline eGFR, history of hypertension, diabetes mellitus, or CVD, change in sCrea from baseline to diagnosis, AKI stage, proteinuria, body mass index, CRP, IGFBP7, TIMP2, and [IGFBP7]*[TIMP2]. For in-hospital mortality, only sFlt-1, CD138, sVCAM-1, IGFBP7, TIMP2, and [IGFBP7]*[TIMP2] were included as independent variables due to the low number of events, which limits the stability of multivariable models and the reliability of effect estimates. To account for heterogeneity of AKI pathophysiology, additional multivariable logistic regression models were constructed including AKI etiology as covariates. Because the number of patients within individual etiologic categories was limited, only the three major AKI mechanisms—postoperative, nephrotoxic, and perfusion-related AKI—were included. To minimize overfitting, these analyses were performed using a parsimonious set of clinically relevant covariates, including age, sex, baseline eGFR, and AKI severity. Receiver Operating Curves (ROC) were used to compare different models. Differences between ROCs were evaluated using the DeLong test.

References

1. Mikacenic C, Hahn WO, Price BL et al. Biomarkers of Endothelial Activation Are Associated with Poor Outcome in Critical Illness. PloS one 2015; 10:e0141251.
2. Greco M, Palumbo C, Sicuro F, Lobreglio G. Soluble Fms-Like Tyrosine Kinase-1 Is A Marker of Endothelial Dysfunction During Sepsis. J Clin Med Res 2018; 10: 700–706.
3. Shapiro NI, Schuetz P, Yano K et al. The association of endothelial cell signaling, severity of illness, and organ dysfunction in sepsis. Crit Care 2010; 14: R182.
4. Uchimido R, Schmidt EP, Shapiro NI. The glycocalyx: a novel diagnostic and therapeutic target in sepsis. Crit Care 2019; 23: 16.
5. Dolmatova E V., Wang K, Mandavilli R, Griendling KK. The effects of sepsis on endothelium and clinical implications. Cardiovasc Res 2020; 117: 60-73.
6. Mansour SG, Zhang WR, Moledina DG et al. The Association of Angiogenesis Markers With Acute Kidney Injury and Mortality After Cardiac Surgery. Am J Kidney Dis 2019; 74: 36–46.
7. Bucaloiu ID, Kirchner HL, Norfolk ER, Hartle JE, Perkins RM. Increased risk of death and de novo chronic kidney disease following reversible acute kidney injury. Kidney Int 2012; 81: 477–485.
8. Pannu N, James M, Hemmelgarn B, Klarenbach S. Association between AKI, recovery of renal function, and long-term outcomes after hospital discharge. Clin J Am Soc Nephrol 2013; 8: 194–202.
9. Meersch M, Schmidt C, Van Aken H et al. Urinary TIMP-2 and IGFBP7 as early biomarkers of acute kidney injury and renal recovery following cardiac surgery. PloS one 2014; 9:e93460.
