# Supplementary material for: Endothelial biomarkers predict kidney and cardiovascular outcomes after acute kidney injury
Source: Clin Kidney J. 2026 Aug 13;19(9):sfag277. doi: 10.1093/ckj/sfag277 (PMC13542715; doi:10.1093/ckj/sfag277)
Supplement: Supplementary file 1 [file sfag277_supplemental_files.zip › Supplementary Tables.pdf]

Supplementary Tables

Vascular signals in AKI: endothelial biomarkers predict renal and cardiovascular outcomes after injury

Running head: Endothelial biomarkers as vascular signals in AKI

Giovana S. Di Marco[1], Katrin Beul[1]; Eike Bormann[2], Hermann Pavenstädt[1], Marcus Brand[1]

[1]Department of Internal Medicine D, University Hospital Muenster, Münster, Germany.

[2]Institute for Biostatistics and Clinical Research, University Hospital Münster, Muenster, Germany.

Corresponding author: Giovana S. Di Marco, Albert-Schweitzer-Campus 1, Gebäude A14, 48149 Münster, Germany. Phone: +49 251 8356911; E-mail: giodimarco@gmail.com; https://orcid.org/0000-0002-1877-638X

Table of Contents:

Table S1: Distribution of causes of uni- and multifactorial AKI stratified by serum sFlt-1 tertiles.
Table S2: Reasons for hospitalization according to serum sFlt-1 tertiles.
Table S3: Patients' characteristics according to AKI stages.
Table S4: Patient outcomes according to AKI stages.
Table S5: Full multivariable logistic regression model for renal nonrecovery at ≥90 days after AKI diagnosis corresponding to Table 7.

Table S1: Distribution of causes of uni- and multifactorial AKI stratified by serum sFlt-1 tertiles.

	**Total**	**Tertiles of sFlt-1 (pg/ml)**			
		**First**	**Second**	**Third**	***P*-Value**[1]
Total	n=93	n=31	n=31	n=31	
Unifactorial AKI	n=24 (26)	n=7 (23)	n=10 (32)	n=7 (23)	
Multifactorial AKI	n=69 (74)	n=24 (77)	n=21 (68)	n=24 (77)	
Causes of unifactorial AKI					
Perfusion-related AKI[2]	1 (1)	0 (0)	1 (3)	0 (0)	1.000
Cardiorenal syndrome	1 (1)	0 (0)	1 (3)	0 (0)	1.000
Hepatorenal syndrome	2 (2)	0 (0)	0 (0)	2 (6)	.326
Nephrotoxic AKI[3]	8 (9)	3 (10)	5 (16)	0 (0)	.091
Glomerular/ interstitial AKI[4]	2 (2)	1 (3)	1 (3)	0 (0)	1.000
Obstruction	4 (4)	2 (6)	0 (0)	2 (6)	.541
Postoperative-AKI	5 (5)	1 (3)	2 (6)	2 (6)	1.000
Inflammation-associated AKI[5]	1 (1)	0 (0)	0 (0)	1 (3)	1.000
**Causes of multifactorial AKI**[6]					
Perfusion-related AKI[2]	51 (74)	16 (67)	16 (76)	19 (79)	.621
Cardiorenal syndrome	13 (19)	4 (17)	4 (19)	5 (21)	1.000

Hepatorenal syndrome	4 (6)	2 (8)	0 (0)	2 (8)	.538
Nephrotoxic AKI[3]	26 (38)	9 (38)	9 (43)	8 (33)	.782
Rhabdomyolysis	2 (3)	2 (8)	0 (0)	0 (0)	.325
Glomerular/ interstitial AKI[4]	2 (3)	2 (8)	0 (0)	0 (0)	.325
Hypercalcemia	2 (3)	0 (0)	2 (10)	0 (0)	.090
Obstruction	2 (3)	0 (0)	1 (5)	1 (4)	.754
Postoperative-AKI	27 (39)	9 (38)	11 (52)	7 (29)	.288
Inflammation-associated AKI[5]	14 (20)	5 (21)	5 (24)	4 (17)	.930

Categorical variables are shown as counts and percentages [n (%)]. [1]P-values were obtained using Fisher's exact test. [2]AKI occurring in the setting of reduced effective renal blood flow, including hypovolemia, hypotension, and renal hypoperfusion; [3]AKI associated with exposure to nephrotoxic drugs. [4]AKI due to acute interstitial nephritis or glomerulonephritis. [5]AKI temporally associated with systemic inflammatory conditions after exclusion of purely postrenal causes. [6]Multifactorial AKI was defined as AKI resulting from two or more concurrent contributing factors.

Table S2: Reasons for hospitalization according to serum sFlt-1 tertiles.

		Tertiles of sFlt-1 (pg/ml)			
	**Total**	**First**	**Second**	**Third**	***P*-Value**[1]
Total	n=93	n=31	n=31	n=31	
Clinical groups					.1095
Cardiovascular/Vascular	37 (40)	15 (48)	10 (32)	12 (39)	
Infectious/Inflammatory	10 (11)	6 (19)	2 (6)	2 (6)	
Gastrointestinal/Hepatobiliary	12 (13)	1 (3)	3 (10)	8 (26)	
Neurological	11 (12)	2 (6)	6 (19)	3 (10)	
Surgical/Trauma/Urological	23 (25)	7 (23)	10 (32)	6 (19)	
Time from hospital admission until enrollment, *in days*	4 (2-8)	5 (2-9)	4 (2-8)	4 (1-9)	.8164

Reasons for hospitalization were categorized according to ICD-10 blocks into five major clinical groups: cardiovascular/vascular (I10-I79, I20-I52), infectious/inflammatory (A00-B99, J00-J22), gastrointestinal/hepatobiliary (K20-K92), neurological (I60-I69, G40-G99), and surgical/trauma/urological causes (S00-S99, N00-N39, T80-T88, Z96). Classification was based on the primary diagnosis leading to hospital admission. Categorical variables are shown as counts and percentages [n (%)]. Numerical data are presented as median (interquartile range). [1]P-values were obtained using Fisher's exact or Kruskal–Wallis test, as appropriate.

Table S3: Patients' characteristics according to AKI stages.

	Total	AKI Stage 1	AKI Stage 2	AKI Stage 3	***P*-Value**[1]
Total	n=93	n=56	n=23	n=14	
Endothelial biomarkers					
sFlt-1 (n=93)	201 (20-1036)	199.5 (22-911)	65 (10-838)	395.5 (121-1284)	.3849
CD138 (n=93)	68 (42-103)	57.5 (34.5-93.5)	64 (42-140)	82 (70-146)	.146
sVCAM-1 (n=93)	1402 ± 630	1251 ± 563	1492 ± 517	1858 ± 824	.003
Renal biomarkers					
Urine IGFPB7[4]	242 (163-385)	192 (148-316)	325.5 (207-569)	360 (270.5-889)	<.001
Urine TIMP2[4]	7 (4-10)	6 (4-9)	8 (5-9)	11 (7-15)	.0130
[IGFPB7]*[TIMP2][4]	1.7 (0.8-4.4)	1.1 (0.6-2.6)	2.7 (1.2-6.3)	3.85 (1.6-14.7)	.002
Patient demographics					
Age	69 (61-78)	71 (62-78.5)	68 (59-78)	66 (44-75)	.315
Sex					.017
Female	41 (44)	18 (32)	14 (61)	9 (64)	
Male	52 (56)	38 (68)	9 (39)	5 (36)	
Clinical data					
CVD	46 (49)	27 (48)	13 (57)	6 (43)	.669
Diabetes	34 (37)	20 (36)	8 (35)	6 (43)	.908
Hypertension	64 (69)	43 (77)	10 (43)	11 (79)	.015
DBP[2]	67 ± 14	70 ± 14	65 ± 13	61 ± 14	.057
SBP[2]	120 ± 20	122 ± 21	121 ± 21	113 ± 17	.334
BMI	25 (23-27)	25.95 (23.5-27.45)	25.1 (23.4-27.1)	24.45 (22.3-26.7)	.507
Hb	11 ± 3	11 ± 3	10 ± 2	9 ± 2	.115
CRP	4.2 (1.6-10.1)	3.7 (1.5-9.1)	4.7 (1.3-10.3)	8.0 (4.1-13.05)	.140
Leukocytes	9.7 (7.0-12.2)	9.39 (6.8-11.48)	10.8 (8.2-13.22)	11.53 (7.96-14.30)	.150
BUN[3]	36 (26-53)	29.5 (22-44)	38 (29-45)	63 (55.5-87)	<.001
Proteinuria[4]	0.6 (0.3-1.7)	0.48 (0.23-1.36)	1.06 (0.52-1.74)	1.77 (0.60-2.73)	.014
Baseline eGFR	58 ± 24	60 ± 21	64 ± 26	43 ± 28	.029
Diagnosis eGFR	29 (18-39)	35.5 (27.5-45.5)	22 (15-30)	11.5 (10-15)	<.001
Baseline sCrea	1.2 (0.9-1.5)	1.2 (0.95-1.0)	1.0 (0.0-1.4)	1.65 (1.3-2.2)	.009
Diagnosis sCrea	2.1 (1.6-3.0)	1.8 (1.5-2.15)	2.60 (2.1-3.3)	4.15 (3.7-4.6)	<.001

Causes of AKI					
Perfusion-related AKI[5]	52 (56)	31 (55)	12 (52)	9 (64)	.764
Cardiorenal syndrome	14 (15)	8 (14)	5 (22)	1 (7)	.455
Hepatorenal syndrome	6 (6)	4 (7)	1 (4)	1 (7)	1.000
Nephrotoxic AKI[6]	34 (37)	21 (38)	7 (30)	6 (43)	.716
Rhabdomyolysis	2 (2)	2 (4)	0 (0)	0 (0)	1.000
Glomerular/interstitial AKI[7]	4 (4)	1 (2)	2 (9)	1 (7)	.220
Hypercalcemia	2 (2)	1 (2)	0 (0)	1 (7)	.339
Obstruction	6 (6)	5 (9)	0 (0)	1 (7)	.403
Postoperative-AKI	32 (34)	20 (36)	8 (35)	4 (29)	.952
Inflammation-associated AKI[8]	15 (16)	10 (18)	5 (22)	0 (0)	.206
Multifactorial AKI[9]	69 (74)	40 (71)	17 (74)	12 (86)	.668
Medication					
ACE inhibitors	25 (27)	17 (30)	5 (22)	3 (21)	.752
AT1 blockers	21 (23)	13 (23)	6 (26)	2 (14)	.732
NSAIDs	10 (11)	9 (16)	1 (4)	0 (0)	.181
Ca^{2+} antagonists	30 (32)	22 (39)	6 (26)	2 (14)	.155
Beta blockers	51 (55)	28 (50)	13 (56)	10 (71)	.371
Diuretics	51 (55)	31 (55)	13 (56)	7 (50)	.914

Numerical data are presented as mean ± SD for normally distributed variables or as median (IQR) for non-normally distributed variables. Categorical variables are shown as counts and percentages [n (%)]. [1]P-values were obtained using ANOVA, Kruskal–Wallis, or Fisher's exact test, as appropriate. [2]One value is missing (n=92); [3]Twenty-three values are missing (n=70); [4]Four urine samples are unavailable (n=89), two of the four patients with missing urine samples had AKI stage 3 and were anuric; the others had AKI stages 2 and 1. [5]AKI occurring in the setting of reduced effective renal blood flow, including hypovolemia, hypotension, and renal hypoperfusion; [6]AKI associated with exposure to nephrotoxic drugs. [7]AKI due to acute interstitial nephritis

or glomerulonephritis. [8]AKI temporally associated with systemic inflammatory conditions after exclusion of purely postrenal causes. [9]Sixty-nine patients presented with two or more concurrent contributing causes (multifactorial AKI). Abbreviations and units: ACEi, angiotensin-converting enzyme inhibitors; Age, in years; AT_1, angiotensin receptor 1, BMI, body mass index (kg/m^2); BUN, blood uren nitrogen (mg/dl); CD138, syndecan-1 (pg/ml); sCrea, serum creatinine (mg/dl); CRP, C-reactive protein (mg/dl); CVD, cardiovascular disease; DBP, diastolic blood pressure (mmHg); sFlt-1, soluble Fms-like tyrosine kinase-1 (pg/ml); eGFR, estimated glomerular filtration rate (ml/min/1.73m^2); IGFBP7; urinary insulin-like growth factor-binding protein 7 (ng/mg creatinine); [IGFBP7]*[TIMP-2] ((ng/mL)^2/1,000); Leukocytes, thousands per microliter; NSAIDs, nonsteroidal anti-inflammatory drugs; Proteinuria, mg protein/mg creatinine; SBP, systolic blood pressure (mmHg); TIMP2, urinary tissue inhibitor of metalloproteinase 2 (ng/mg creatinine); sVCAM, soluble vascular cell adhesion molecule (ng/ml). All urine and serum samples for biomarker determination were collected at the time of AKI diagnosis.

Table S4: Patient outcomes according to AKI stages.

	Total	AKI Stage 1	AKI Stage 2	AKI Stage 3	*P*-Value[1]
Total	n=93	n=56	n=23	n=14	
Change in sCrea	0.8 (0.5-1.4)	0.6 (0.45-0.7)	1.3 (1.1-1.8)	2.5 (1.8-2.8)	<.001
Duration of AKI	5 (3-8)	4.5 (2.5-7.5)	5 (3-10)	8 (7-17)	.005
AKI ≥ 7 days	35 (38)	17 (30)	7 (30)	11 (79)	.040
Length of hospitalization	9 (5-15)	8 (4-16)	8 (6-14)	13.5 (8-22)	.056
In-hospital death	7 (8)	5 (9)	2 (9)	0 (0)	.733
Renal nonrecovery[2]	40 (43)	23 (46)	9 (43)	5 (57)	.752
Follow-up eGFR[2]	49 ± 23	52 ± 20	53 ± 25	32 ± 22	.010
New-onset CVD	33 (35)	21 (38)	9 (39)	3 (21)	.532
Length of follow-up[2]	5 ± 2	6 ± 2	5 ± 2	5 ± 2	.775

Numerical data are presented as mean ± SD for normally distributed variables or as median (IQR) for non-normally distributed variables. Categorical variables are shown as counts and percentages [n (%)]. [1]P-values were obtained using ANOVA, Kruskal–Wallis, or Fisher's exact test, as appropriate. [2]Outcomes assessed in survivors (n=85) ≥ 90 days after AKI diagnosis. Abbreviations and units: sCrea, serum creatinine (mg/dl); CVD, cardiovascular disease; Duration of AKI, in days; sFlt-1, soluble Fms-like tyrosine kinase-1 (pg/ml); Length of follow-up, in days; eGFR, estimated glomerular filtration rate (ml/min/1.73m^2); Length of hospitalization, in days. All urine and serum samples for biomarker determination were collected at the time of AKI diagnosis.

Table S5: Full multivariable logistic regression model for renal nonrecovery at ≥90 days after AKI diagnosis corresponding to Table 7.

Predictor	OR (95% CI)	*P*-Value
AKI mechanisms		
Perfusion-related AKI	1.072 (0.325-3.529)	0.9095
Nephrotoxic AKI	1.948 (0.563-6.745)	0.2925
Postoperative AKI	0.702 (0.203-2.435)	0.5776
Endothelial biomarkers		
sFlt-1	1.212 (1.060-1.386)	**0.0048**
CD138	0.949 (0.882-1.022)	0.1650
sVCAM-1	1.000 (0.999-1.001)	0.7269
Clinical covariates		
Age	1.027 (0.982-1.073)	0.2481
Sex	0.756 (0.222-2.577)	0.6547
Baseline eGFR	1.043 (1.009-1.079)	**0.0131**
AKI Stage		0.2032
AKI Stage 3 vs Stage 1	0.223 (0.036-1.383)	
AKI Stage 3 vs Stage 2	0.196 (0.025-1.514)	

Values are presented as adjusted odds ratios (ORs) with 95% confidence intervals (CIs) for all predictors included in the model. AKI, acute kidney injury; CD138, syndecan-1; sFlt-1, eGFR, estimated glomerular filtration rate; soluble Fms-like tyrosine kinase-1. sVCAM-1, soluble vascular cell adhesion molecule-1.
